# Targeting copper death genotyping associated gene RARRES2 suppresses glioblastoma progression and macrophages infiltration

**DOI:** 10.1186/s12935-023-02950-6

**Published:** 2023-05-29

**Authors:** Tao Yan, He Yang, Yun Meng, Huadong Li, Qing Jiang, Junsi Liu, Caixia Xu, Yanpeng Xue, Jiayi Xu, Yan Song, Xiaojie Chu, Lijuan Wang, Xin Chen, Fengyuan Che

**Affiliations:** 1 Central Laboratory, Linyi People’s Hospital, Guangzhou University of Chinese Medicine, Linyi, 276000 Shandong Province China; 2 Linyi Key Laboratory of Neurophysiology, Linyi People’s Hospital, Linyi, 276000 Shandong Province China; 3 Department of Neurosurgery, First Affiliated Hospital of Harbin Medical University, Harbin, 150001 Heilongjiang Province China; 4 Key Colleges and Universities Laboratory of Neurosurgery in Heilongjiang Province, Harbin, 150001 Heilongjiang Province China; 5 Institute of Neuroscience, Sino-Russian Medical Research Center, Harbin Medical University, Harbin, 150001 Heilongjiang Province China; 6 Department of Neurosurgery, Linyi People’s Hospital, Linyi, 276000 Shandong Province China; 7 Department of Nuclear Medicine, The Fourth Hospital of Harbin Medical University, Harbin, 150001 Heilongjiang Province China; 8 Department of Clinical Pharmacy, Daqing Oilfield General Hospital, Daqing, 163001 Heilongjiang Province China; 9 Department of Hematology, Linyi People’s Hospital, Guangzhou University of Chinese Medicine, Linyi, 276000 Shandong Province China; 10 Department of Neurology, Linyi People’s Hospital, Guangzhou University of Chinese Medicine, Linyi, 276000 Shandong Province China

**Keywords:** Glioblastoma, Cuproptosis, RARRES2, IDH status, Immune cell infiltration

## Abstract

**Background:**

Copper homeostasis is associated with malignant biological behavior in various tumors. The excessive accumulation of copper can induce tumor death, which is named cuproptosis, and it is also closely related to tumor progression and the formation of the immune microenvironment. However, the associations of cuproptosis with glioblastoma (GBM) prognosis and microenvironment construction are poorly understood.

**Method:**

First, TCGA and GEO (GSE83300, GSE74187) merged datasets were used to analyze the association of cuproptosis-related genes (CRGs) with GBM. Then, we performed cluster analysis of CRGs in GBM from the GEO (GSE83300, GSE74187) and TCGA merged datasets. Subsequently, the prognostic risk model was constructed by least absolute shrinkage and selection operator (LASSO) according to gene expression features in CRG clusters. Next, we performed a series of in-depth analyses, including tumor mutational burden (TMB) analysis, cluster analysis, and GBM IDH status prediction. Finally, RARRES2 was identified as a target gene for GBM treatment, especially IDH wild-type GBM. In addition, we further analyzed the correlation of CRG clusters and RARRES2 expression with the GBM immune microenvironment by ESTIMATE and CIBERSORT analyses. In vitro experiments were conducted to demonstrate that targeting RARRES2 inhibits glioblastoma progression and macrophage infiltration, particularly IDH wild-type GBM.

**Results:**

In the present study, we demonstrated that the CRG cluster was closely related to GBM prognosis and immune cell infiltration. Moreover, the prognostic risk model constructed with the three genes (MMP19, G0S2, RARRES2) associated with the CRG clusters could well evaluate the prognosis and immune cell infiltration in GBM. Subsequently, after further analyzing the tumor mutational burden (TMB) in GBM, we confirmed that RARRES2 in the prognostic risk model could be used as a crucial gene signature to predict the prognosis, immune cell infiltration and IDH status of GBM patients.

**Conclusion:**

This study fully revealed the potential clinical impact of CRGs on GBM prognosis and the microenvironment, and determined the effect of the crucial gene (RARRES2) on the prognosis and tumor microenvironment construction of GBM, meanwhile, our study also revealed over-expressed RARRES2 is related to the IDH satus of GBM, which provides a novel strategy for the treatment of GBM, particularly IDH wild-type GBM.

**Supplementary Information:**

The online version contains supplementary material available at 10.1186/s12935-023-02950-6.

## Introduction

Glioblastoma (GBM) is a rare brain tumor with a high fatality rate; the 5-year mortality rate is more than 90% [1]. Even after undergoing the standard Stupp treatment protocol, the median survival time of GBM patients is less than 2 years [2]. Although various therapeutic strategies, including chemoradiotherapy and immunotherapy, are still being developed and applied, the prognosis of GBM patients remains unsatisfactory [1]. In addition, the high heterogeneity within GBM and the lack of specific target genes are major obstacles limiting the success of GBM therapy. Therefore, there is an urgent need to explore new biomarkers and therapeutic targets for this refractory tumor.

Copper is a cofactor of some crucial enzymes that perform physiological functions [3]; its abnormal accumulation produces toxic effects on organisms [4]. Peter Tsvetkov et al. reported that copper ions can directly bind to lipoylated tricarboxylic acid cycle (TCA) proteins to induce cell death, and this form of cell death is named cuproptosis [5]. Recently, some studies confirmed that the deficiency or overload of copper in the body is clearly associated with many diseases, such as hereditary diseases, Wilson’s disease (WD), Alzheimer's disease (AD) and cardiovascular diseases [6]. Similarly, researchers have found abnormal copper metabolism in a variety of tumors, including breast, thyroid and prostate cancers [7]. Copper also promotes tumor progression by inducing epithelial–mesenchymal transition (EMT) and angiogenesis [7-9]. Moreover, copper homeostasis can affect tumor epigenetic modifications at the level of chromatin modifications and transcription factors to favor tumor progression [10]. Recently, copper was shown to regulate the expression of programmed death ligand 1 (PD-L1) in tumors, which allows tumor cells to evade immune surveillance [11]. Meanwhile, some studies have identified that copper controls the mitogenic signaling pathway, thereby promoting oncogenesis [8]. The oncogenic roles of copper-dependent lysyl coxidase enzymes (LOX and LOXL1-4) have been demonstrated in various tumors, including colorectal cancer, hepatocellular carcinoma and breast cancer [12-15]. These evidences suggest that abnormal copper metabolism is a major cause of tumor pathogenesis, and increasing evidence has recognized that tumor cells have a higher demand for copper relative to most other tissues, which indicates that copper metabolic vulnerability can be an alternative for tumor treatment [16]. Therefore, the in-depth exploration of copper metabolism in tumors may become an effective strategy for anticancer therapies.

Investigation of the role of copper metabolism in glioma progression is still in progress. Qian et al. demonstrated that copper overload in astroglioma cells is associated with reactive oxygen species (ROS) production [17]. Copper chelators can induce the cytotoxicity of copper oxide nanoparticles (CuO-NPs) by blocking the accumulation of copper in C6 cells [18]. Wang et al. reported that the copper-associated gene STEAP2 is involved in glioma prognosis [19]. These above evidences indicate that abnormal copper metabolism may be involved in the occurrence and development of glioma.

The effectiveness of traditional copper ionophores and copper chelators as antitumor agents has been confirmed; however, these remedies lack selectivity [20]. Moreover, the existence of tumor heterogeneity results in different tumors having different metabolic characteristics [21]; thus, there may also be differences in copper metabolism in tumor cells, suggesting that the development of specific targets for tumor copper metabolism has the potential to be a driving factor for the success of tumor therapy. In the present study, GBM patients were first divided into two subgroups by clustering analysis based on CRGs expression, and then further analysis confirmed that RARRES2 is a potential target for GBM treatment. We also analyzed the correlations of RARRES2 expression with GBM patient prognosis, IDH status and immune cell infiltration. Ultimately, we confirmed that RARRES2 overexpression was negatively correlated with GBM patient prognosis and that the RARRES2 expression level could predict the IDH status of GBM patients. Moreover, RARRES2 overexpression was correlated with the formation of an immunosuppressive microenvironment in GBM patients. Therefore, these observations indicated that targeting RARRES2 may provide a new therapeutic strategy to improve GBM prognosis, particularly IDH wild-type GBM.

## Methods and materials

### Data acquisition

The transcriptome and survival data of 168 glioblastoma (GBM) and 5 normal brain tissues were derived from The Cancer Genome Atlas (TCGA, https://portal.gdc.cancer.gov/). The microarray and overall survival (OS) information of 110 GBM samples were obtained from GSE74187 (60 GBM samples) and GSE83300 (50 GBM samples) in the Gene Expression Omnibus (GEO, Home—GEO—NCBI (nih.gov)) database. The IDH status and transcriptome data of 342 GBM samples were downloaded from the Chinese Glioma Genome Atlas (CGGA, Home | CGGA—Chinese Glioma Genome Atlas).

### Cluster and principal component analysis (PCA)

GBM samples were grouped by cluster analysis according to typing-related gene expression. First, the expression of typing-related genes in GBM patients was obtained by the R package limma, and then ConsensusClusterPlus in R was used to perform cluster analysis. PCA was performed using R packages (limma, ggplot2) according to the expression of typing-related genes and cluster data of GBM patients.

### GSVA, GO, and KEGG enrichment analyses

The GBM transcriptome data were obtained from the TCGA and GEO databases. The cluster data of GBM patients were derived from cluster analysis. Then, three R packages (GSEABase, GSEABase, GSVA) were utilized for subsequent GSVA analysis, and the heatmap of GSVA was drawn by R software. GO and KEGG enrichment analyses were performed by R packages (clusterProfiler, org.Hs.eg.db, enrichplot, ggplot2).

### Prognostic risk model construction

The construction of the prognostic risk model was carried out using R packages (survival, caret, glmnet, survminer, and timeROC). In GBM patients, we obtained the expression data of prognosis-related genes as well as survival-related information. Least absolute shrinkage and selection operator (LASSO) was performed to construct the prognostic risk model for GBM patients. Finally, the prognostic risk model was constructed by three genes (MMP19, G0S2, and RARRES2).

### Immune correlation analysis

The R package ESTIMATE was used to evaluate the tumor microenvironment (TME) scores of each GBM sample, and then the correlation of the TME scores with the prognostic risk model and risk genes was graphed by the R packages reshape2 and ggpubr. The R package (CIBERSORT) was used to analyze the state of immune cell infiltration. Then, R packages (limma, reshape2, tidyverse, ggplot2, ggpubr and ggExtra) were utilized to analyze the results of CIBERSORT. The transcriptome data were obtained to further determine the relationship between RARRES2 and immune checkpoints, and the relationship was analyzed using R packages (limma, reshape2, ggplot2, ggpubr, and corrplot).

### Survival prognosis, univariate Cox and receiver operating characteristic (ROC) analyses

First, the patients’ survival time and grouping information were acquired. Subsequently, the R packages (survival and survminer) were used to perform survival prognosis analysis and to draw the diagram of the relationship between grouping and survival. R packages (limma and survival) were used for univariate Cox analysis to assess the relationship between the expression of prognosis-related genes and the survival time of GBM patients; ultimately, the correlation of prognosis-related genes with survival time was acquired. To further explore the correlation of patient survival with grouping data, ROC curves were drawn by the R package (survivalROC).

### Tumor mutational burden (TMB) analysis

The gene mutation information of GBM was derived from the TCGA database. Then, the GBM patients were divided into high- and low-risk subgroups according to the prognostic risk score. Next, the gene mutation information of GBM patients was matched with every patient in the high- and low-risk subgroups. Then, the R package maftools was utilized to calculate the TMB of GBM patients.

### Cell culture

The glioblastoma cell lines (U251 and LN229) and U937 monocytes were obtained from the Laboratory of Neurosurgery, the First Affiliated Hospital of Harbin Medical University. Dulbecco’s modified Eagle’s medium (DMEM; Gibco, USA) containing 10% fetal bovine serum (FBS FND500, Excell bio, Australia) was used to culture U251 and LN229 cell lines at 37 °C in 5% CO_2_. RPMI-1640 medium (RPMI-1640; Sigma, USA) containing 10% FBS was used to culture U937 cells. U937 cells were induced to transform into macrophages with 100 ng/mL phorbol 12-myristate 13-acetate (PMA, MCE) culture for 24 h.

### MTT assay

A total of 3–4 × 10^3^ cells were seeded in 96-well plates and cultured for 24 h. Then, siRARRES2 was used to culture cells for 48 h; after treatment, 10 μl MTT (5 mg/ml) was added to each well, and the cells were incubated at 5% CO_2_ and 37 °C for 4 h. Next, the medium was discarded, and 150 μl of DMSO was added. A BioTek ELx800 (USA) microplate reader was used to evaluate cell viability at 490 nm.

### EdU assay

The U251 and LN229 glioma cell lines were seeded in 6-well plates and cultured with siRARRES2 for 48 h. Then, an EdU assay kit (Beyotime, China) was used to measure the effect of siRARRES2 on glioma proliferation ability according to the instructions.

### Colony formation assay

Glioma cells were seeded in 6-well plates and incubated at 5% CO_2_ and 37 °C for 24 h. Next, siRARRES2 was used to culture glioma cells. Then, the 6-well plate was cultured with DMEM containing 10% FBS; 10 days later, the colonies were fixed with methanol and stained with 0.1% crystal violet (Beyotime).

### Western blotting

Protein samples were obtained from normal brain tissue and IDH(Mut) and IDH(WT) GBM tissues. Next, the BCA Protein Assay kit (Beyotime) was used to determine the protein concentrations. Then, the protein lysates were separated by SDS‒PAGE electrophoresis. Then, the proteins were transferred to PVDF membranes and blocked in 5% skim milk followed by overnight incubation with primary antibodies at 4 °C. The membranes were rinsed and incubated with secondary antibodies for 1 h at room temperature. The GeneGnome XRQ Imaging System (Syngene, UK) was used to observe immunoreactivity. The following primary antibodies were used in this study: anti-RARRES2 (Cat#10216–1-AP Proteintech China) and anti-β-actin (Cat#66009–1-Ig Proteintech China).

### Cell transfection and qRT‒PCR

RARRES2 siRNAs were purchased from Genial Biosystems (China) and transfected into glioma cell lines using Lipofectamine 8000 (Cat# C0533, Beyotime, China) according to the manufacturer’s instructions. Next, TRIzol reagent (Cat#T9424, Sigma, USA) was used to collect the total RNA, and the Roche Transcriptor cDNA Synthesis Kit (Cat#4897030001, Roche, Switzerland) was used to obtain cDNA. Finally, SYBR Green PCR Master Mix (Cat#4913914001, Roche, Switzerland) and an ABI Prism 7500 fast thermocycler (Applied Biosystems, CA, USA) were used to assess the expression of RARRES2. The primer sequences are listed in Table 1.

**Table 1:** Sequence of siRNA, prismer

siRNA sequence	
siNC	5′UUCUCCGAACGUGUCACGUTT3′
RARRES2–si#1	5′GGAAGAAACCCGAGUGCAATT3′
RARRES2–si#2	5′AGGUGGCCCUGGAGGAAUUTT3′
RARRES2–si#3	5′CCAUAGAGACCCAAGUUCUTT3′
Prismer sequence	
GAPDH	F-5′ GCACCGTCAAGGCTGAGAAC3′, R-5′TGGTGAAGACGCCAGTGGA3′
RARRES2	F-5′CAGGAGACCAGTGTGGAGAG3′, R-5′CTCAGAGCCCAGTTTGATGC3′

### Transwell assay

U251 and LN229 cell lines were transfected with siNC/siRARRES2 for 48 h, and the supernatants were collected; then, the supernatants were mixed with RPMI-1640 at a volume ratio of 1:1 to obtain siNC-derived conditioned medium (siNC CM) and siRARRES2-derived conditioned medium (siRARRES2 CM). Next, 1 × 10^5^ macrophages were seeded in the upper chamber, and different CM types were added to the lower chamber for 24 h. After culture, the upper chamber was detached, and then the number of infiltrating macrophages was assessed by Transwell assays (Cat#TCS003024: JET BIOFIL, China).

### Statistical analysis

R version 4.2.0, Perl, and SPSS software were used for bioinformatics analysis, and the R packages used in the present study were obtained from http://bioconductor.org/. Student’s t test or one-way analysis of variance (Prism software version 8.02) was used to compare the differences in data between groups in vitro experiments. P values less than 0.05 were considered statistically significant in our research.

## Results

### Cuproptosis-related genes (CRGs) are involved in glioblastoma

**Fig. 1:**
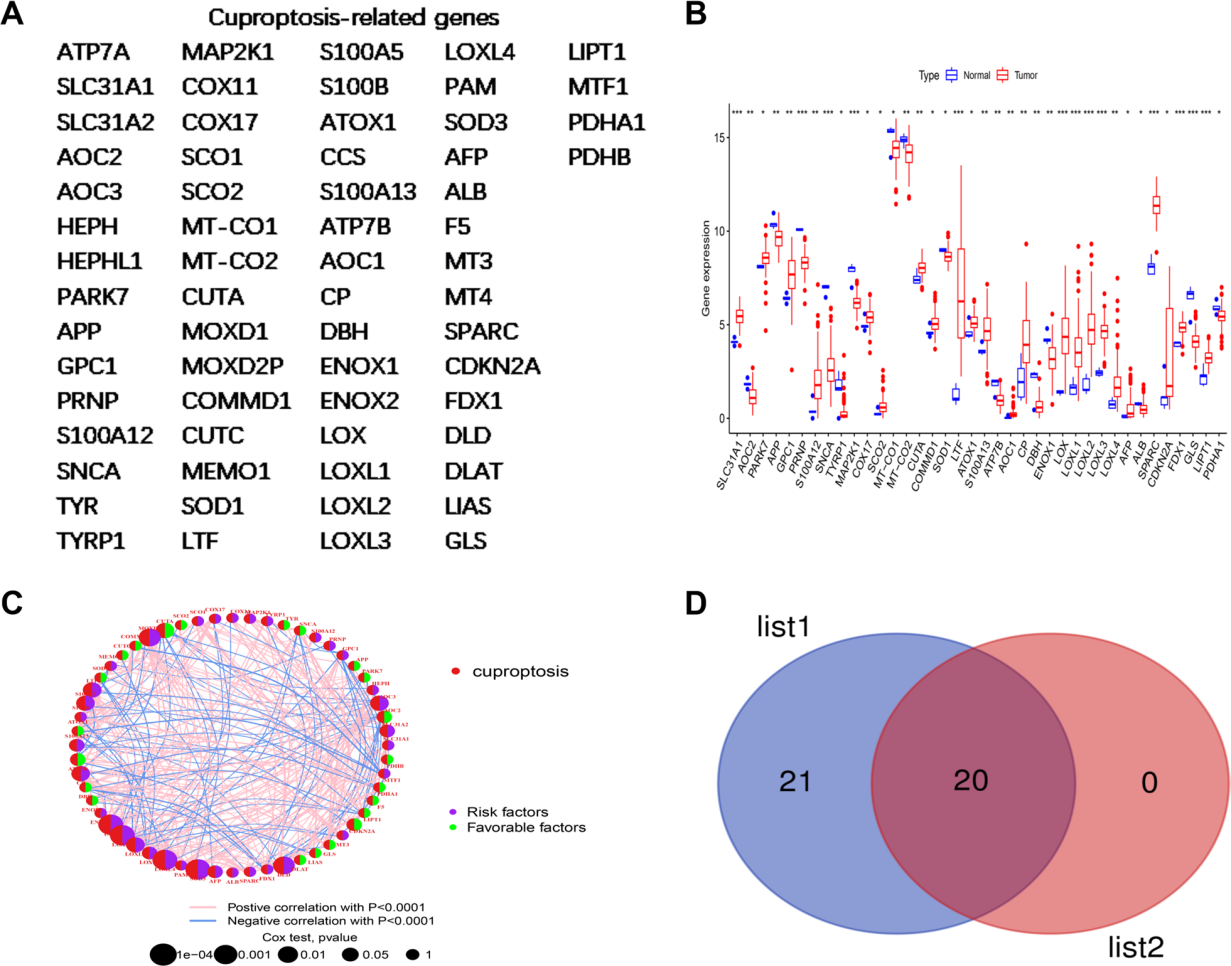
Summary of cuproptosis-related genes in GBM **A** The list of 64 cuproptosis-related genes (CRGs). **B** The expression levels of CRGs in GBM tissue relative to normal brain tissue in the TCGA database. **C** Prognostic network diagram revealing the correlation of CRGs with GBM prognosis. (Risk factors: high-risk genes; Favorable factors: low-risk genes; The size of the dot represents the P value) **D** The CRGs associated with survival and CRGs associated with the hazard ratio (HR) in GBM were cross-analyzed with a Venn diagram. List 1: CRGs associated with GBM survival. List 2: CRGs associated with the HR

To explore the correlation of CRGs with GBM, 64 CRGs were obtained from published articles [22, 23] (Fig. 1A). Then, The Cancer Genome Atlas (TCGA) database was utilized to quantify the expression of CRGs in GBM. Most CRGs were differentially expressed in GBM (Fig. 1B), preliminarily suggesting that copper metabolism was abnormally regulated in GBM. To further confirm the relationship between 64 CRGs and GBM, the Gene Expression Omnibus (GEO) (GSE83300, GSE74187) and TCGA datasets were merged, and the associations of 64 CRGs with survival and the hazard ratio (HR) in GBM patients were further analyzed. Ultimately, 21 CRGs related to survival time and 20 CRGs associated with HR were identified in GBM. Subsequently, further intersection analysis observations indicated that 20 CRGs were significantly associated with GBM survival and HR risk (Fig. 1C–D, Additional file 1: Figure S1A–C). Taken together, these observations revealed that copper metabolism was closely associated with GBM prognosis, and the correlation warrants further investigation.

### Cluster analysis of CRGs in GBM

**Fig. 2:**
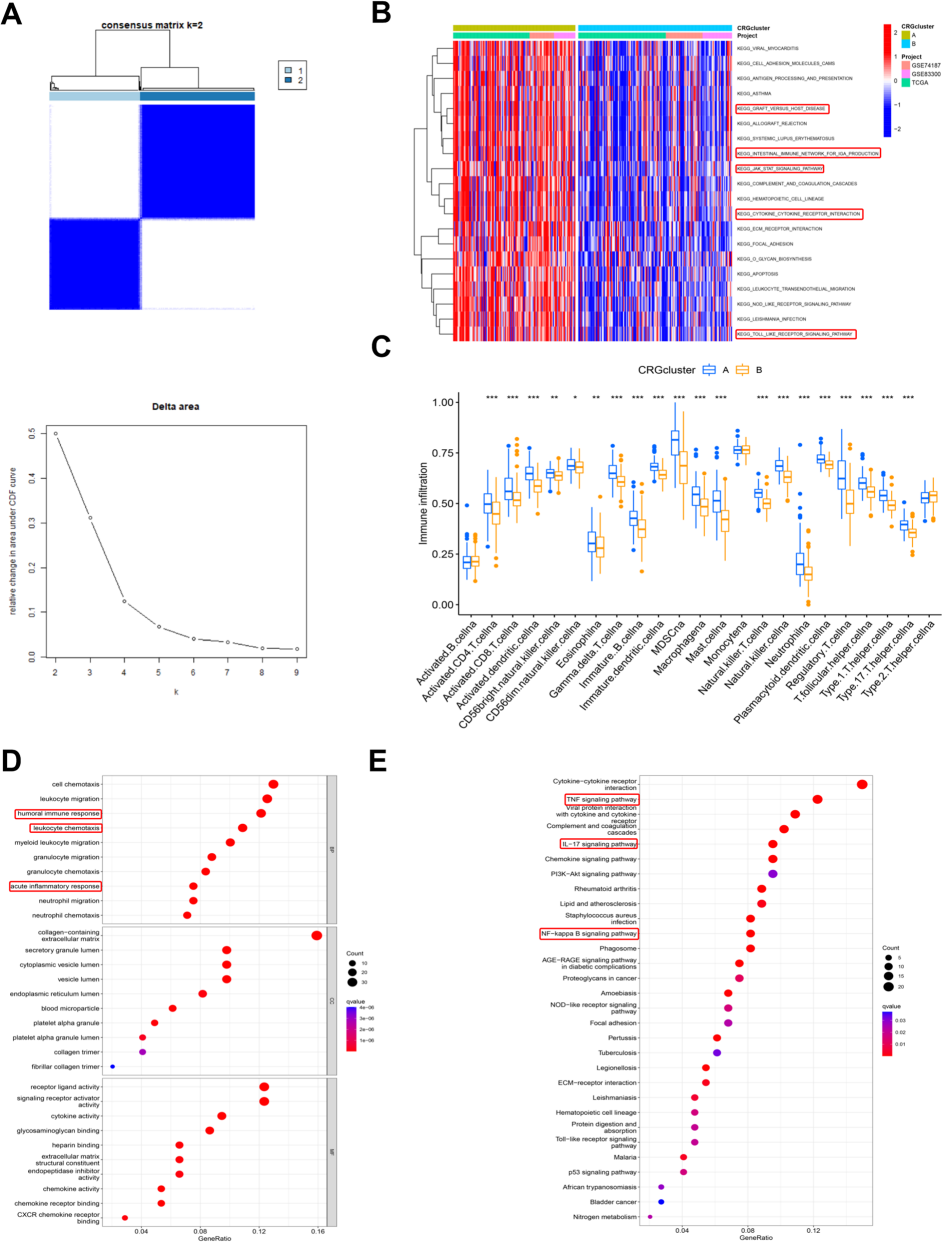
Cluster analysis of cuproptosis-related genes in GBM. **A** The CRG cluster analysis divided the GBM patients into two subgroups (k = 2) according to the transcriptome of CRGs. **B** Gene set variation analysis (GSVA) revealed that there were distinct differences in KEGG pathway enrichment between CRG cluster A and B. **C** The correlation of immune cell infiltration with the CRG clusters. **D**–**E** GO analysis and KEGG analysis revealed the physiological functions and pathways affected by the differentially expressed genes between CRG cluster A and B

To further elucidate the correlation of CRGs with GBM, the datasets merged by GEO (GSE83300, GSE74187) and TCGA were subjected to cluster analysis of GBM according to CRG expression. Finally, the GBM patients were divided into two subgroups (Fig. 2A). The principal component analysis (PCA) results showed that there was good demarcation between CRG cluster A and B (Additional file 2: Figure S2A). The heatmap of CRG expression suggested that CRG expression significantly differed between CRG cluster A and B (Additional file 2: Figure S2B). Moreover, subsequent Kaplan‒Meier survival analysis revealed that the survival time of GBM patients in CRG cluster A was significantly lower than that in CRG cluster B (Additional file 2: Figure S2C). These results indicated that the cluster analysis dividing GBM patients into two subgroups according to CRG expression has high accuracy in evaluating the prognosis of GBM patients.

To determine the differences in physiological functions between CRG cluster A and B, gene set variation analysis (GSVA) was used to perform Kyoto Encyclopedia of Genes and Genomes (KEGG) enrichment analysis of CRG cluster A and B. The GSVA results suggested that immune-related pathways, including graft-versus-host disease, intestinal immune network, JAK-STAT signaling pathway, cytokine‒cytokine receptor interaction and Toll-like receptor signaling pathway, were obviously enriched, indicating that immune-associated physiological functions were different in groups A and B (Fig. 2B). Then, we further analyzed the difference in immune cell infiltration in CRG cluster A and B and found that there were significant differences in infiltrating immune cells between CRG cluster A and B (Fig. 2C). These results supported that there are differences in the immune response between CRG cluster A and B. To further confirm these differences between CRG cluster A and B, 252 differentially expressed genes (DEGs) were screened between CRG cluster A and B (Additional file 2: Figure S2D). Then, Gene Ontology (GO) analysis and KEGG analysis were carried out to evaluate the physiological functions and pathways affected by the 252 DEGs. The GO results revealed that immune-related physiological functions, such as the humoral immune response, leukocyte chemotaxis and acute inflammatory response, were markedly regulated by the DEGs. The KEGG analysis results suggested that immune-related pathways, including the TNF signaling pathway, IL-17 signaling pathway and NF-kappa B signaling pathway, were strongly affected by the DEGs (Fig. 2D–E). All of the above findings further indicated that the immune response is obviously different between CRG cluster A and B.

### Prognostic and molecular features of the DEGs in the CRG cluster

Given that 252 DEGs were identified in the above CRG clusters, univariate Cox analysis was performed to screen the genes associated with prognosis from 252 DEGs. Finally, 210 genes associated with GBM prognosis were captured (Table 2). To investigate the molecular features of these 210 genes, further cluster analysis results showed that the GBM patients could be divided into another two clusters (gene cluster A and gene cluster B) according to the expression of the 210 genes (Additional file 3: Figure S3A–B). Subsequent analysis showed that the prognosis of patients in gene cluster A was worse than that of patients in gene cluster B (Additional file 3: Figure S3C). These results again indicated that these 210 genes are closely involved in the prognosis of GBM patients.

**Table 2:** The uniCox analysis of DRGs between A and B in CRG cluster

Id	HR	HR.95L	HR.95H	pvalue
LTF	1.063594	1.022523	1.106315	0.002151
NAMPT	1.185164	1.081909	1.298274	0.000259
CHI3L1	1.100668	1.043475	1.160995	0.000427
ABCC3	1.121298	1.04432	1.203949	0.001605
SOD2	1.133381	1.028249	1.249263	0.011708
CP	1.11934	1.034108	1.211598	0.005272
C1R	1.182455	1.062368	1.316117	0.002161
C1S	1.150388	1.041924	1.270142	0.005558
CCL2	1.139336	1.052889	1.232879	0.001195
C1RL	1.281327	1.146314	1.432243	1.28E-05
FCGR2A	1.22779	1.060353	1.421667	0.006081
CHI3L2	1.090677	1.02866	1.156432	0.003661
DPYD	1.304922	1.132344	1.503803	0.000236
ANXA1	1.221504	1.112316	1.341411	2.81E-05
CD44	1.181375	1.063384	1.312459	0.001905
NFKBIZ	1.35733	1.202622	1.531939	7.49E-07
SAA1	1.091059	1.046709	1.137289	3.85E-05
TREM1	1.1924	1.096331	1.296888	4.03E-05
PTX3	1.220903	1.131558	1.317303	2.64E-07
F13A1	1.094078	1.018782	1.174939	0.013457
MAP3K8	1.200151	1.060654	1.357995	0.003803
ICAM1	1.242385	1.108685	1.392209	0.000187
BCL3	1.305501	1.158409	1.471271	1.24E-05
VASN	1.300224	1.172577	1.441767	6.37E-07
PLAUR	1.314771	1.168368	1.47952	5.54E-06
ANXA2	1.214313	1.090873	1.351722	0.000385
BCL7A	0.813881	0.710197	0.932702	0.003056
TNFSF14	1.334853	1.16721	1.526573	2.46E-05
CD163	1.145974	1.056584	1.242927	0.001008
CSTA	1.198727	1.065704	1.348354	0.002525
BIRC3	1.291411	1.18134	1.411739	1.84E-08
TLR2	1.194512	1.047264	1.362464	0.008098
CFI	1.191548	1.077127	1.318124	0.000668
THBD	1.275986	1.138612	1.429935	2.75E-05
SOCS3	1.204485	1.09223	1.328276	0.000193
MARCO	1.123876	1.042769	1.21129	0.002244
MOXD1	1.157305	1.070507	1.251141	0.00024
CFB	1.27222	1.123177	1.44104	0.000152
S100A4	1.204281	1.075061	1.349032	0.001328
LILRB3	1.282625	1.132447	1.452718	8.94E-05
SAA2	1.14604	1.06677	1.2312	0.000193
VDR	1.374487	1.212153	1.55856	7.04E-07
IL2RA	1.121881	1.022628	1.230766	0.014957
SERPING1	1.195025	1.058392	1.349295	0.004027
HES5	0.891487	0.832897	0.954199	0.000927
STEAP3	1.236529	1.11381	1.372769	6.86E-05
GPX8	1.214231	1.099607	1.340804	0.000125
AQP9	1.159905	1.063195	1.265413	0.000839
LOX	1.241847	1.137362	1.355931	1.36E-06
FCGBP	1.084889	1.002582	1.173953	0.042967
CLEC5A	1.259374	1.124384	1.41057	6.70E-05
BHLHE40	1.308793	1.139558	1.50316	0.000139
LILRB2	1.186457	1.050825	1.339595	0.005773
IBSP	1.165636	1.083999	1.253421	3.52E-05
CD109	1.194975	1.056061	1.35216	0.004727
TNFAIP2	1.281658	1.146763	1.432421	1.22E-05
SERPINA1	1.145718	1.011947	1.297173	0.031758
THBS1	1.195523	1.07592	1.32842	0.000898
TYMP	1.256974	1.103401	1.431921	0.000582
EMP3	1.234035	1.103975	1.379417	0.000215
BATF	1.194857	1.049973	1.359733	0.006947
MMP19	1.272197	1.135781	1.424996	3.18E-05
CCL7	1.175929	1.082905	1.276943	0.000116
TIMP1	1.206517	1.111553	1.309594	7.17E-06
S100A10	1.137897	1.027264	1.260445	0.013308
ADAM8	1.27296	1.13467	1.428105	3.90E-05
S100A8	1.106923	1.026917	1.193162	0.007957
CLCF1	1.317452	1.161498	1.494347	1.80E-05
PLOD2	1.214787	1.077376	1.369724	0.001489
MAN1C1	1.138622	1.009837	1.283831	0.034022
STAB1	1.154321	1.012789	1.315631	0.031525
ANXA2P1	1.178977	1.04627	1.328518	0.006885
EFEMP1	1.119908	1.020958	1.228448	0.016421
ALOX5AP	1.158436	1.018964	1.316998	0.02464
CCL20	1.188585	1.084946	1.302124	0.000206
MMD2	0.842694	0.762798	0.930958	0.000758
CXCL5	1.143567	1.041149	1.25606	0.005074
MSR1	1.200286	1.054789	1.365852	0.005622
FLRT1	0.843472	0.754356	0.943116	0.002809
DUSP26	0.874459	0.800239	0.955563	0.003033
DPYSL4	0.895996	0.803904	0.998638	0.04719
S100A9	1.092888	1.010515	1.181976	0.026311
PLAU	1.18014	1.070478	1.301037	0.000873
SLITRK1	0.875191	0.793259	0.965586	0.007854
PDPN	1.188493	1.088917	1.297174	0.00011
IL6	1.179965	1.056626	1.317701	0.003306
CXCL3	1.146868	1.024458	1.283904	0.017333
GRID2	0.835011	0.758206	0.919595	0.00025
TNFRSF11B	1.243946	1.11488	1.387954	9.40E-05
C21orf62	1.130459	1.011329	1.263622	0.030909
LY96	1.175206	1.040722	1.32707	0.009223
ZDHHC22	0.826201	0.731886	0.932669	0.002021
SLC16A3	1.304344	1.145923	1.484667	5.78E-05
RARRES1	1.231426	1.114896	1.360136	4.06E-05
DDIT4L	1.171282	1.076807	1.274045	0.000229
FAM20A	1.251979	1.108332	1.414244	0.000301
CXCL2	1.188379	1.071288	1.318269	0.00111
MYBPH	1.1201	1.035716	1.211358	0.004538
RIPPLY2	0.806761	0.722251	0.901159	0.000143
RDH10	1.166757	1.044436	1.303404	0.006345
CXCL14	1.069651	1.006352	1.136931	0.030509
CXCL6	1.182539	1.083016	1.291207	0.000186
TDO2	1.147325	1.043715	1.261221	0.004427
LGALS3	1.200855	1.095141	1.316772	9.90E-05
MAST1	0.863938	0.767549	0.972432	0.015388
SRPX2	1.124544	1.035281	1.221503	0.005408
COL6A2	1.140745	1.055888	1.232422	0.000841
GBP1	1.126152	1.020484	1.242761	0.018112
NEU4	0.846666	0.778305	0.921031	0.000107
NKAIN1	0.881784	0.809922	0.960024	0.003725
LYZ	1.128475	1.032003	1.233965	0.008029
TAGLN	1.151788	1.01893	1.301969	0.02383
MAOB	1.177147	1.071395	1.293338	0.000684
PLA2G5	1.129388	1.024115	1.245482	0.014799
PLIN2	1.200387	1.066931	1.350535	0.002387
ANPEP	1.222744	1.102045	1.356661	0.000149
SOX8	0.843356	0.768795	0.925148	0.000309
CXCL1	1.194391	1.105366	1.290584	6.96E-06
RPRM	0.864919	0.790686	0.946122	0.001526
SERPINA5	1.201554	1.090182	1.324303	0.000216
TGFBI	1.123718	1.029512	1.226544	0.009027
FN1	1.236588	1.112112	1.374997	8.75E-05
GDAP1L1	0.84383	0.770681	0.923923	0.000242
SPOCD1	1.095753	1.010088	1.188683	0.027692
TCTEX1D1	1.118628	1.025316	1.220432	0.011652
RUNDC3A	0.867527	0.780838	0.963839	0.008154
CNTFR	0.885287	0.806479	0.971796	0.010425
CYP1B1	1.147323	1.043385	1.261616	0.004561
IGFBP3	1.122194	1.037309	1.214024	0.004069
ANGPTL4	1.153476	1.059868	1.25535	0.000945
COL8A1	1.138543	1.055896	1.227659	0.000739
ARSJ	1.17166	1.062025	1.292611	0.001575
OSM	1.134416	1.03332	1.245403	0.008092
PLEK2	1.214934	1.080747	1.365782	0.001113
SERPINE1	1.216777	1.091554	1.356365	0.000399
DLL3	0.911315	0.858977	0.966842	0.002088
GNG4	0.871284	0.782932	0.969607	0.011546
OLIG2	0.821978	0.762736	0.885821	2.80E-07
ADM	1.182667	1.08669	1.28712	0.000102
CXXC4	0.863995	0.782908	0.953479	0.003645
DLL1	0.881728	0.805222	0.965503	0.006567
GZMA	1.136681	1.017419	1.269923	0.023493
DSCAML1	0.899477	0.828596	0.976421	0.011415
SEZ6L	0.875798	0.80224	0.9561	0.003047
SPP1	1.246436	1.12775	1.377613	1.60E-05
CFH	1.22217	1.096873	1.36178	0.000278
CA12	1.0953	1.002523	1.196663	0.043824
HMX1	0.884398	0.818342	0.955786	0.001924
PDLIM4	1.198118	1.102772	1.301707	1.94E-05
SHD	0.888667	0.825607	0.956543	0.001672
HES6	0.843943	0.775957	0.917886	7.51E-05
SLC16A10	1.21746	1.088061	1.362247	0.000599
PTGS2	1.168318	1.056069	1.292497	0.00254
CELF3	0.906928	0.833105	0.987292	0.02412
PHACTR3	0.903629	0.830234	0.983513	0.019048
COL1A1	1.114787	1.041843	1.192838	0.001648
PCDH15	0.835282	0.753959	0.925375	0.000573
G0S2	1.198988	1.11618	1.287939	6.69E-07
SLC47A2	1.098077	1.018722	1.183614	0.0145
CCL8	1.10118	1.005207	1.206317	0.038304
LIF	1.175926	1.059129	1.305604	0.002395
EPHB1	0.902668	0.830224	0.981433	0.016439
OLIG1	0.791304	0.723712	0.865208	2.77E-07
NEUROD1	0.833776	0.756978	0.918364	0.000227
NKAIN4	0.860151	0.791549	0.934698	0.000382
MYT1	0.89705	0.81277	0.99007	0.030911
LOXL1	1.267488	1.149674	1.397375	1.92E-06
CSPG5	0.878245	0.800695	0.963307	0.005914
FMOD	1.101605	1.032516	1.175316	0.003409
COL3A1	1.098676	1.024908	1.177752	0.00796
TNFRSF12A	1.210943	1.092322	1.342446	0.000274
VIPR2	0.870821	0.794712	0.954218	0.003034
GBP3	1.154083	1.052366	1.265631	0.002333
RAB3C	0.887917	0.805644	0.978592	0.016568
DIRAS3	1.190047	1.084126	1.306316	0.000254
AEBP1	1.211556	1.112101	1.319905	1.13E-05
KCNB1	0.880635	0.797758	0.972121	0.011713
HSPA6	1.149308	1.045904	1.262936	0.003816
FREM3	0.785641	0.70131	0.880113	3.12E-05
COL5A1	1.192842	1.083028	1.31379	0.000345
C7orf57	1.123767	1.030484	1.225494	0.008312
OCIAD2	1.144323	1.046809	1.250922	0.003011
NXPH1	0.899929	0.837353	0.967182	0.004138
LUM	1.121057	1.026272	1.224597	0.011235
KLRC3	0.847093	0.767727	0.934664	0.000946
COL1A2	1.156327	1.066668	1.253522	0.00042
MMP7	1.107547	1.034637	1.185594	0.003282
CXCL13	1.110406	1.022472	1.205902	0.01285
POSTN	1.069681	1.019547	1.12228	0.005953
VSTM2B	0.914898	0.838912	0.997768	0.04438
GADD45G	0.902015	0.829584	0.98077	0.015751
ACTG2	1.13345	1.033874	1.242617	0.007585
RGS1	1.10955	1.019714	1.207301	0.015816
ATCAY	0.913822	0.837087	0.997591	0.044025
RARRES2	1.212841	1.124831	1.307737	5.15E-07
CA10	0.910068	0.848552	0.976045	0.008316
SMOC1	0.908219	0.852072	0.968065	0.003108
VEGFA	1.163134	1.069814	1.264595	0.000398
CCL18	1.083258	1.022405	1.147733	0.006706
TSTD1	1.11194	1.025927	1.205165	0.009792
METTL7B	1.108521	1.020878	1.203688	0.014219
BCAN	0.897478	0.834275	0.96547	0.003695
MMP9	1.163675	1.075107	1.259539	0.000175
GPR17	0.9086	0.847619	0.973969	0.00685
CA9	1.112531	1.033126	1.198038	0.004764
CA3	1.086855	1.021506	1.156385	0.008476
CXCL10	1.084947	1.002839	1.173778	0.042299
DAPL1	0.919885	0.852769	0.992282	0.030742
NR0B1	0.906163	0.8443	0.972558	0.00631
CNGA3	1.086672	1.008911	1.170428	0.028226

**Fig. 3:**
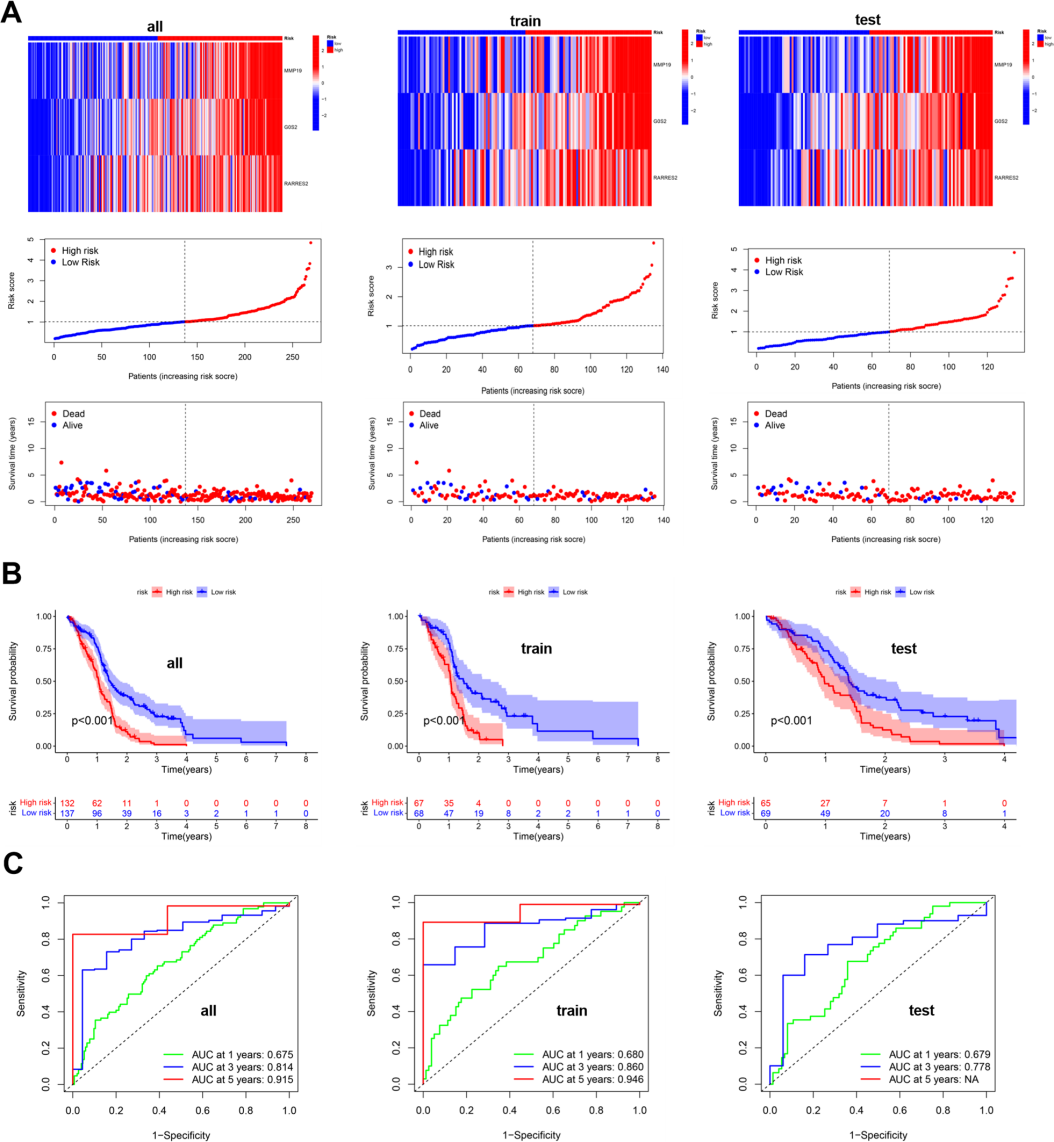
Prognostic risk model establishment and evaluation. **A** The prognostic risk model revealed the correlations of the prognostic risk score with survival status and prognostic gene expression in all, training and test subgroups. **B** Kaplan‒Meier survival analysis revealed the correlation of the prognostic risk score with GBM patient survival time in all, training and test subgroups. **C** ROC analysis to predict the survival rates at 1, 3, and 5 years according to the prognostic risk score in all, training and test subgroups

Therefore, the 210 DEGs were used to establish the prognostic risk model. Finally, three genes (MMP19, G0S2, RARRES2) were screened to construct the prognostic risk model in GBM by least absolute shrinkage and selection operator (LASSO) regression (Additional file 4: Figure S4A, Table 3). Then, the correlation of the CRG clusters and gene clusters with the risk score of the prognostic risk model was explored, and we found that the CRG clusters and gene clusters were associated with the risk score of the prognostic risk model (Additional file 4: Figure S4B–C). Next, the Sankey diagram quantified the relationship between the CRG clusters, gene clusters, prognostic risk score and prognostic results (Additional file 4: Figure S4D). Subsequently, the GBM patients were divided into three groups (all, train and test) to evaluate the accuracy and feasibility of the prognostic risk model as a predictor of GBM prognosis. The following analysis showed that in the above three groups (all, train and test), the expression of MMP19, G0S2 and RARRES2 in the high-risk group was higher than that in the low-risk group, and the risk scores of patients in the high-risk group were increased. Moreover, we found that the number of nonsurviving patients also increased as the patient's risk score increased. Meanwhile, Kaplan‒Meier survival analysis suggested that the survival time of patients in the high-risk group was decreased compared to that in the low-risk group (Fig. 3A–B). The receiver operating characteristic (ROC) curve revealed that the area under the curve (AUC) values were all greater than 0.7 or close to 0.7 in evaluating the survival outcome of GBM patients (1, 3, and 5 years) (Fig. 3C), indicating that the high expression of the three genes is associated with the poor prognosis of GBM patients. Overall, these findings suggested that the prognostic risk model established by three genes (MMP19, G0S2, RARRES2) can serve as a predictor of GBM prognosis.

**Table 3:** The risk scores of GBM patients in the prognostic risk model

Id	Futime	Fustat	MMP19	G0S2	RARRES2	RiskScore	Risk
TCGA-06–0171	1.093151	1	5.257944	6.966346	5.400603	1.980711	High
TCGA-32–2634	1.89863	0	2.868419	3.455889	6.365254	0.781102	Low
TCGA-12–3652	2.909589	1	2.893743	1.610738	4.446743	0.461497	Low
TCGA-32–2638	2.09863	1	3.766394	3.847878	2.5896	0.619739	Low
TCGA-06–0882	1.731507	1	2.854485	5.236664	5.766722	0.950283	Low
TCGA-06–0168	1.638356	1	3.019028	5.87246	7.406848	1.340242	High
TCGA-32–2616	0.613699	1	2.209315	5.945457	6.980835	1.079542	High
TCGA-41–2572	1.112329	1	3.233816	2.583397	3.718774	0.525547	Low
TCGA-06–0750	0.076712	1	5.135422	5.401198	7.09469	1.881494	High
TCGA-06–5859	0.380822	0	1.335037	4.561041	8.245411	0.849976	Low
TCGA-16–0846	0.326027	1	1.735178	3.035217	4.319167	0.441932	Low
TCGA-06–0178	7.345205	1	2.663793	0.440533	1.976275	0.267058	Low
TCGA-06–0211	0.986301	1	3.57368	4.454643	5.385024	0.934867	Low
TCGA-06–0139	0.991781	1	5.938662	8.37904	7.705789	3.831067	High
TCGA-06–0644	1.052055	1	5.313856	5.221879	5.90201	1.631571	High
TCGA-06–2569	0.035616	0	1.474509	4.424322	2.714162	0.421925	Low
TCGA-32–1970	1.282192	1	1.500257	6.848799	6.572587	1.012666	Low
TCGA-27–2523	1.339726	1	1.781657	5.479403	6.706372	0.884981	Low
TCGA-28–5213	0.816438	0	5.286111	7.521383	8.117673	3.076224	High
TCGA-06–0130	1.079452	1	4.796698	7.716768	4.931028	1.898215	High
TCGA-06–0125	3.967123	1	4.504572	4.421246	3.914656	0.939792	Low
TCGA-27–1835	1.775342	1	1.329017	5.868615	1.819282	0.456086	Low
TCGA-27–2524	0.632877	1	4.248445	6.546408	7.205134	1.885213	High
TCGA-27–1837	1.169863	1	2.581836	4.232985	3.446934	0.570052	Low
TCGA-41–5651	1.260274	1	2.237901	6.139437	6.769168	1.089317	High
TCGA-28–5216	1.136986	0	5.670306	4.712124	4.217682	1.311102	High
TCGA-12–5295	1.243836	1	2.174808	3.330782	5.780637	0.612894	Low
TCGA-14–0736	1.260274	1	2.738343	5.763385	5.603968	0.985002	Low
TCGA-06–2559	0.410959	1	5.090959	5.805544	7.10863	1.987676	High
TCGA-06–2558	1.041096	1	3.186766	5.095352	5.907433	1.016288	High
TCGA-76–4928	0.257534	1	4.989599	6.6577	6.131428	1.958159	High
TCGA-06–2562	1.046575	1	3.863747	7.446471	6.63257	1.85489	High
TCGA-06–5412	0.378082	1	4.989987	8.49064	5.596005	2.42853	High
TCGA-06–0646	0.479452	1	3.715486	7.033421	5.860659	1.526902	High
TCGA-15–0742	1.147945	1	2.935547	4.362418	6.689032	0.950442	Low
TCGA-19–2619	0.805479	0	3.510802	4.776556	3.924719	0.804027	Low
TCGA-12–0618	1.082192	1	2.811666	4.785979	5.799262	0.881824	Low
TCGA-32–4213	1.654795	0	4.567654	3.757583	4.628078	0.941823	Low
TCGA-32–2615	1.328767	1	4.513157	6.765201	6.241632	1.8239	High
TCGA-28–2514	0.438356	0	2.533559	5.076098	3.775761	0.670563	Low
TCGA-02–0047	1.227397	1	5.371345	5.266732	2.247593	1.041164	High
TCGA-27–2519	1.506849	1	3.603351	2.883832	5.534385	0.751935	Low
TCGA-06–0747	0.224658	1	1.648994	3.33564	2.829935	0.375541	Low
TCGA-41–2571	0.071233	1	3.662341	5.787008	6.65064	1.377321	High
TCGA-06–5414	0.747945	0	3.015062	5.983528	5.002084	1.000953	Low
TCGA-06–0645	0.479452	1	5.125399	7.90626	4.821156	2.067636	High
TCGA-06–5410	0.29589	1	4.865582	8.325993	6.737991	2.668701	High
TCGA-06–5413	0.734247	0	4.150181	5.515786	6.832703	1.500374	High
TCGA-06–1804	1.134247	1	1.301984	6.129597	7.855174	1.023532	High
TCGA-19–5960	1.246575	1	1.278753	2.576634	9.473555	0.722938	Low
TCGA-06–0878	0.59726	0	4.817306	7.515394	6.697243	2.317601	High
TCGA-76–4927	1.465753	1	3.876466	5.34976	5.232519	1.123451	High
TCGA-19–0957	1.824658	1	4.46454	5.092389	6.086482	1.3657	High
TCGA-02–2483	1.276712	0	3.633336	3.858672	1.318864	0.512681	Low
TCGA-28–2509	0.39726	0	3.464377	5.729674	7.006543	1.36969	High
TCGA-06–2563	2.553425	0	2.115406	2.57331	2.233035	0.34154	Low
TCGA-06–0158	0.90137	1	1.780229	5.351727	4.347319	0.641046	Low
TCGA-76–4929	0.30411	1	3.807896	4.559378	8.222668	1.437131	High
TCGA-14–1402	2.671233	1	2.513508	3.888652	5.159209	0.663343	Low
TCGA-32–1982	0.389041	1	2.77067	4.766365	5.331147	0.820728	Low
TCGA-06–0686	1.183562	1	3.728717	5.510954	4.520153	1.018656	High
TCGA-06–5858	0.512329	0	3.213701	4.998163	5.989656	1.017554	High
TCGA-06–2561	1.471233	1	5.325107	4.670277	5.431919	1.41379	High
TCGA-27–1832	0.821918	1	4.932678	4.893378	7.78477	1.819509	High
TCGA-14–1825	0.635616	1	2.540316	3.040677	4.515064	0.538691	Low
TCGA-06–0238	1.109589	1	2.799472	4.621288	3.184218	0.613194	Low
TCGA-06–5856	0.312329	1	3.071021	6.284832	4.832705	1.03858	High
TCGA-06–0152	1.027397	1	3.786045	3.995332	5.114042	0.879999	Low
TCGA-06–0184	5.824658	1	2.545752	3.575637	4.840382	0.610845	Low
TCGA-06–5416	0.558904	0	1.806065	3.54771	6.956739	0.681129	Low
TCGA-06–0141	0.857534	1	5.014745	6.666301	6.77057	2.139637	High
TCGA-06–5418	0.227397	1	4.201204	6.012994	6.279101	1.525995	High
TCGA-02–2485	1.287671	0	1.419986	6.167334	6.211019	0.855186	Low
TCGA-19–1390	2.115068	1	4.001883	3.48546	4.817024	0.819761	Low
TCGA-12–5299	0.268493	1	3.656195	5.006615	6.454266	1.188642	High
TCGA-06–0138	2.019178	1	3.188797	5.309839	6.215843	1.093448	High
TCGA-26–5135	0.739726	1	3.517512	6.24963	2.817701	0.87767	Low
TCGA-06–2557	0.090411	1	2.037452	5.687983	4.15831	0.696385	Low
TCGA-08–0386	1.50137	1	2.929035	3.771394	9.023344	1.168217	High
TCGA-19–2620	0.405479	1	4.105256	4.317138	5.593634	1.053036	High
TCGA-28–5209	1.210959	0	2.028116	4.907852	5.05511	0.690944	Low
TCGA-14–1034	1.328767	1	4.093182	5.08687	5.412108	1.156065	High
GSM1912920	2.805479	1	1.830694	6.810575	6.528342	1.074226	High
GSM1912921	2.405479	1	2.911416	3.397975	2.339976	0.466358	Low
GSM1912922	0.975342	1	2.591612	3.432993	5.94971	0.695583	Low
GSM1912925	1.610959	1	3.979364	4.179839	5.947595	1.050104	High
GSM1912928	1.945205	0	4.087136	4.173429	5.774005	1.049877	High
GSM1912930	1.057534	1	2.96321	6.160099	6.058228	1.164853	High
GSM1912932	1.134247	1	4.154816	3.937574	5.417385	0.981081	Low
GSM1912939	1.093151	1	1.430111	5.211108	9.578396	1.138026	High
GSM1912945	1.517808	1	3.655826	7.036139	6.561693	1.649999	High
GSM1912946	0.854795	1	3.525116	6.867616	7.240492	1.705333	High
GSM1912947	1.542466	1	4.874994	8.830492	6.374698	2.759796	High
GSM1912949	0.073973	1	2.948701	4.93561	5.953982	0.947925	Low
GSM1912950	0.542466	1	6.031132	7.102411	6.325029	2.686464	High
GSM1912952	3.191781	0	2.780092	3.599622	4.433198	0.611817	Low
GSM1912954	2.032877	1	2.32825	5.889954	7.532912	1.178358	High
GSM1912955	1.084932	1	4.572788	6.494665	6.839715	1.91265	High
GSM1912958	1.441096	1	4.390108	6.195699	6.629811	1.709594	High
GSM1912960	2.254795	0	4.788958	7.597871	7.575903	2.611373	High
GSM1912963	0.994521	1	5.398642	6.733762	6.063398	2.143546	High
GSM1912965	0.99726	1	3.067773	5.498776	6.183187	1.092587	High
GSM1912966	3.572603	0	1.912948	4.618217	2.756964	0.480112	Low
GSM1912968	0.39726	1	2.977263	5.510184	7.183985	1.2205	High
GSM1912970	1.553425	1	2.099074	5.683673	7.897202	1.138723	High
GSM1912971	1.134247	1	2.911141	0.884141	2.929987	0.340787	Low
GSM1912972	1.175342	1	5.029757	3.951088	2.896275	0.857937	Low
GSM1912974	0.39726	0	2.552213	5.507256	6.39405	1.006755	Low
GSM1912978	0.70411	1	4.43662	6.070272	8.660262	2.196885	High
GSM1912979	1.624658	1	3.012997	1.422667	2.500343	0.358288	Low
GSM2198607	3.510411	0	2.110133	3.78593	4.238317	0.532272	Low
GSM2198608	1.029863	1	5.055892	6.800586	6.771693	2.204203	High
GSM2198612	3.802192	1	2.681909	2.951886	7.353389	0.787979	Low
GSM2198613	2.599726	0	2.546214	2.350088	3.8287	0.443849	Low
GSM2198614	0.367397	1	3.861435	3.390743	4.832304	0.78548	Low
GSM2198616	1.496712	1	3.91811	7.626207	6.350951	1.861113	High
GSM2198617	3.523562	0	2.391019	4.294238	1.386963	0.424278	Low
GSM2198618	1.034795	1	2.301708	5.955583	6.274419	1.007323	Low
GSM2198619	2.870137	0	1.861793	5.898183	6.128213	0.891965	Low
GSM2198622	0.535068	1	4.332713	7.106829	7.33233	2.127892	High
GSM2198624	2.767397	1	2.418207	6.008797	5.876457	0.989473	Low
GSM2198628	0.656712	1	3.203528	6.34114	5.123024	1.118625	High
GSM2198629	1.070137	1	4.330042	5.665603	6.071302	1.447366	High
GSM2198630	2.942466	1	2.787766	3.359308	6.357249	0.755584	Low
GSM2198632	2.134521	0	2.476014	0.605496	0.463415	0.216798	Low
GSM2198637	0.759452	1	3.500749	4.54496	6.782593	1.116497	High
GSM2198638	1.248493	1	4.259012	4.56213	4.930605	1.03819	High
GSM2198640	0.756986	0	1.712949	4.581533	5.457475	0.646623	Low
GSM2198644	1.483562	1	3.311276	4.693484	3.229273	0.695751	Low
GSM2198645	1.60274	1	2.095265	1.147195	1.383477	0.244552	Low
GSM2198646	1.875616	0	4.23043	3.138715	3.109843	0.655449	Low
GSM2198650	0.810411	1	3.866019	5.0162	4.259695	0.939716	Low
GSM2198651	1.762192	0	3.099012	5.179137	7.692367	1.270204	High
GSM2198653	0.913151	1	2.298002	4.43304	6.79479	0.849966	Low
GSM2198654	1.043014	1	3.188	5.461357	5.416113	1.010169	Low
TCGA-12–1597	1.849315	1	2.186274	1.969769	3.983131	0.395235	Low
TCGA-28–2513	0.608219	0	5.137413	4.509862	5.982129	1.421706	High
TCGA-27–2521	1.39726	1	2.005268	0.48931	1.131281	0.209767	Low
TCGA-28–5208	1.490411	1	2.943648	10.10117	3.937988	1.627282	High
TCGA-06–2567	0.364384	1	3.041452	5.474321	8.674746	1.489607	High
TCGA-06–0744	3.906849	1	1.514921	2.300705	5.24318	0.423603	Low
TCGA-14–0817	0.449315	1	1.917228	5.362063	7.715324	1.0181	High
TCGA-12–3650	0.912329	1	2.286315	3.677263	4.183514	0.539664	Low
TCGA-27–1830	0.421918	1	6.291398	5.015188	4.4853	1.623864	High
TCGA-14–0871	2.410959	1	2.629518	6.678992	6.198826	1.196821	High
TCGA-06–0190	0.868493	1	5.459093	6.76027	7.923659	2.767477	High
TCGA-27–1834	3.378082	1	1.69332	3.535773	7.085436	0.674728	Low
TCGA-06–5417	0.424658	0	2.145936	1.884773	2.637664	0.325458	Low
TCGA-76–4931	0.764384	1	2.964901	5.418043	5.669295	0.988264	Low
TCGA-02–2486	1.693151	1	3.454163	4.701774	5.519866	0.963354	Low
TCGA-14–0789	0.936986	1	6.858069	7.328512	6.9293	3.587243	High
TCGA-14–0790	1.147945	1	1.385504	7.661381	5.247436	0.945572	Low
TCGA-28–5220	1.063014	1	1.183853	4.62801	7.414018	0.747478	Low
TCGA-14–2554	1.457534	1	2.83108	4.203483	6.23191	0.855255	Low
TCGA-06–5408	0.978082	1	3.305313	5.407219	6.781843	1.22368	High
TCGA-06–2570	2.624658	0	3.515613	7.253666	1.609355	0.877829	Low
TCGA-28–1753	0.10137	0	5.7929	4.062634	4.839389	1.318086	High
TCGA-06–2565	1.386301	1	2.141692	1.926503	1.264424	0.274449	Low
TCGA-16–1045	2.419178	1	3.874499	5.669397	4.857678	1.124606	High
TCGA-28–5218	0.430137	1	5.277428	2.209443	10.34081	1.793667	High
TCGA-26–5139	0.131507	0	4.451947	7.040126	5.795932	1.774182	High
TCGA-02–0055	0.208219	1	6.099075	6.57385	9.136727	3.601341	High
TCGA-19–2629	2.019178	1	2.753908	0.667879	1.925469	0.280174	Low
TCGA-06–0743	2.2	1	3.247555	4.704367	5.951324	0.974556	Low
TCGA-06–0157	0.265753	1	2.48334	6.313622	6.074644	1.078898	High
TCGA-12–0616	1.227397	1	2.457011	4.658015	5.850852	0.806749	Low
TCGA-12–0821	0.884932	1	3.406533	4.705513	4.849884	0.875629	Low
TCGA-06–0174	0.268493	1	2.788784	5.394205	6.22081	1.017723	High
TCGA-27–1831	1.383562	1	2.711537	4.725571	5.361975	0.808509	Low
TCGA-06–5411	0.69589	1	2.986565	4.996717	4.168212	0.767335	Low
TCGA-06–2564	0.49589	0	4.73285	5.041383	5.480878	1.327729	High
TCGA-76–4926	0.378082	1	4.351308	3.619629	5.856106	1.030337	High
TCGA-06–0132	2.112329	1	4.123515	5.220026	5.188131	1.154206	High
TCGA-14–0781	0.079452	1	5.826159	4.138316	4.992684	1.369788	High
TCGA-14–0787	0.186301	1	3.271925	4.64413	5.177498	0.8789	Low
TCGA-19–4065	0.586301	0	5.219292	5.653903	5.050949	1.532876	High
TCGA-26–5133	1.238356	0	1.73209	-0.1141	2.477816	0.214175	Low
TCGA-14–1823	1.487671	1	3.253994	6.81314	6.672335	1.483742	High
TCGA-06–0129	2.805479	1	2.502882	0.488865	0.722764	0.221382	Low
TCGA-27–2528	1.315068	1	3.4055	1.192174	6.289661	0.611085	Low
TCGA-26–1442	2.610959	0	1.277298	0.425429	1.859642	0.195181	Low
TCGA-28–5215	0.917808	1	4.846651	4.527773	6.040312	1.349808	High
TCGA-32–1980	0.09863	1	2.754472	5.275392	5.752582	0.934092	Low
TCGA-76–4932	3.994521	1	4.354102	7.121781	7.411618	2.164481	High
TCGA-28–5207	0.939726	1	1.797938	3.69735	7.667878	0.762302	Low
TCGA-06–0210	0.616438	1	5.540655	6.474261	5.371312	1.942368	High
TCGA-32–5222	1.60274	1	2.806682	7.42242	4.077159	1.062561	High
TCGA-06–0749	0.224658	1	1.562071	4.599853	5.100253	0.599786	Low
TCGA-19–2625	0.339726	1	3.912006	4.612753	5.983637	1.111993	High
TCGA-06–0219	0.060274	1	2.434164	4.135398	4.297547	0.606738	Low
TCGA-12–3653	1.210959	1	1.5325	3.094678	5.351349	0.487536	Low
TCGA-26–5132	0.783562	0	2.804655	4.902885	9.059932	1.361795	High
TCGA-06–0187	2.268493	1	3.788407	5.84864	6.799583	1.456028	High
TCGA-26–5136	1.580822	1	2.675159	5.210606	9.313883	1.435248	High
TCGA-41–3915	0.986301	1	5.431622	5.353637	5.92566	1.712819	High
TCGA-19–1787	1.054795	1	2.402762	3.485713	7.541523	0.826009	Low
TCGA-15–1444	4.210959	1	2.694033	2.842073	2.531157	0.418606	Low
TCGA-32–2632	0.736986	1	1.930266	6.638636	7.318591	1.1824	High
TCGA-41–4097	0.016438	1	3.914091	4.340023	4.790876	0.915281	Low
TCGA-27–2526	0.238356	1	2.581937	4.383693	8.460494	1.10958	High
TCGA-06–0649	0.175342	1	4.230896	4.000131	6.1064	1.099837	High
TCGA-28–5204	1.243836	1	2.449252	4.603214	7.41854	0.976348	Low
TCGA-06–0745	0.654795	1	3.375788	6.593571	7.761982	1.692597	High
TCGA-19–1389	0.386301	1	5.675585	4.851391	8.210834	2.237623	High
TCGA-06–0156	0.487671	1	4.795289	6.791279	7.176161	2.1926	High
TCGA-26–5134	0.457534	0	2.667793	5.187708	5.780076	0.907779	Low
TCGA-19–2624	0.013699	1	3.259533	6.214481	4.271663	0.995335	Low
TCGA-14–1829	0.59726	0	4.298785	4.120227	6.561969	1.205218	High
TCGA-28–1747	0.210959	1	2.055726	2.059711	3.676457	0.374729	Low
TCGA-06–0221	1.652055	1	1.868871	1.276958	3.181742	0.299363	Low
TCGA-76–4925	0.4	1	2.826366	4.72911	5.25423	0.817674	Low
TCGA-12–0619	2.909589	1	4.286767	6.208226	6.05404	1.5563	High
GSM1912923	1.8	1	4.431954	5.573979	6.904562	1.622782	High
GSM1912924	0.515068	1	4.014005	5.93201	7.336437	1.658047	High
GSM1912926	3.380822	0	3.010001	4.434134	3.027478	0.610692	Low
GSM1912927	1.054795	1	4.046538	6.466724	3.488958	1.107618	High
GSM1912929	0.479452	1	2.127435	3.556856	5.347846	0.594429	Low
GSM1912931	1.147945	1	3.636654	6.189381	6.596977	1.447851	High
GSM1912933	1.531507	0	1.403147	2.617943	2.91452	0.32232	Low
GSM1912934	3.558904	0	3.276137	4.129284	4.898722	0.783768	Low
GSM1912935	3.854795	1	2.906828	3.558769	7.580976	0.935122	Low
GSM1912936	1.90137	0	4.061133	2.819912	3.069636	0.59862	Low
GSM1912937	0.443836	1	3.607403	2.885273	3.471926	0.577881	Low
GSM1912938	1.753425	1	5.17788	5.062455	4.792617	1.341245	High
GSM1912940	3.005479	0	2.650914	1.174042	3.131316	0.34597	Low
GSM1912941	0.693151	1	4.130245	6.448752	7.248996	1.820818	High
GSM1912942	1.131507	1	3.125673	2.474634	4.527826	0.560146	Low
GSM1912943	2.219178	1	0.979432	4.874464	6.071487	0.625844	Low
GSM1912944	0.890411	1	5.412774	7.230583	6.609518	2.490334	High
GSM1912948	2.632877	0	3.561262	3.068681	3.512546	0.591769	Low
GSM1912951	1.547945	1	1.649167	2.387261	3.016009	0.332088	Low
GSM1912953	0.780822	1	2.984786	5.176914	3.532154	0.726992	Low
GSM1912956	1.380822	1	2.83709	4.820981	2.800975	0.606993	Low
GSM1912957	1.517808	1	4.705658	5.639276	5.91122	1.530211	High
GSM1912959	1.956164	1	5.400761	5.145598	5.929805	1.648495	High
GSM1912961	2.164384	0	2.168335	1.524771	2.431426	0.301219	Low
GSM1912962	1.460274	1	3.408818	5.994417	7.493211	1.500939	High
GSM1912964	1.128767	1	3.612666	6.39079	7.092988	1.583636	High
GSM1912967	1.246575	1	1.772777	6.736841	7.036804	1.119613	High
GSM1912969	1.6	1	5.430521	7.534953	7.101216	2.79091	High
GSM1912973	1.789041	0	3.408094	3.994307	4.646355	0.764374	Low
GSM1912975	0.857534	1	2.811731	4.519818	7.984166	1.119748	High
GSM1912976	2.635616	0	1.250159	1.636214	4.282281	0.319309	Low
GSM1912977	2.263014	1	2.394929	5.39298	6.383525	0.955119	Low
GSM2198606	1.421096	1	4.570737	5.746551	6.112411	1.551122	High
GSM2198609	0.683836	1	4.533149	5.937674	6.854804	1.743168	High
GSM2198610	1.116164	1	2.189965	2.78151	4.074096	0.453787	Low
GSM2198611	2.1	1	5.46253	6.188348	6.364033	2.075477	High
GSM2198615	1.367671	1	2.712125	3.478948	5.355207	0.666081	Low
GSM2198620	1.343014	1	2.462854	2.967024	6.015604	0.634939	Low
GSM2198621	1.089041	1	3.987406	5.413493	1.020061	0.677067	Low
GSM2198623	0.877808	1	5.352616	6.784499	6.014362	2.125907	High
GSM2198625	0.961644	1	1.888849	3.280106	5.40386	0.545089	Low
GSM2198626	2.232329	1	2.287111	4.831587	5.722276	0.786118	Low
GSM2198627	1.807397	0	3.603757	2.100834	4.53022	0.58568	Low
GSM2198631	1.132603	1	4.245437	5.702667	6.227342	1.458501	High
GSM2198633	1.578082	1	5.594837	7.073561	6.836694	2.60159	High
GSM2198634	1.44	1	3.411277	5.433868	7.177962	1.322344	High
GSM2198635	1.431781	1	1.83475	4.35874	6.039353	0.690817	Low
GSM2198636	1.296986	0	2.834183	3.369504	8.108181	0.956639	Low
GSM2198639	3.488219	0	1.598831	4.057856	4.845939	0.538023	Low
GSM2198641	1.52137	1	5.450526	8.78245	6.642274	3.205886	High
GSM2198642	1.527123	1	1.993787	2.720358	2.927427	0.372136	Low
GSM2198643	0.980548	1	4.995782	6.19433	5.485087	1.679881	High
GSM2198647	0.507945	1	3.206289	5.358787	6.97393	1.218752	High
GSM2198648	0.908219	1	2.772583	3.093856	7.123068	0.79736	Low
GSM2198649	1.596986	1	5.824394	9.012817	8.956505	4.841324	High
GSM2198652	2.610411	1	1.776214	5.270479	6.949823	0.88294	Low
GSM2198655	0.77589	1	5.929805	8.388817	7.118578	3.551943	High

### The tumor microenvironment (TME) in high-risk patients with glioblastoma was more inclined to have immunosuppressive phenotypes

**Fig. 4:**
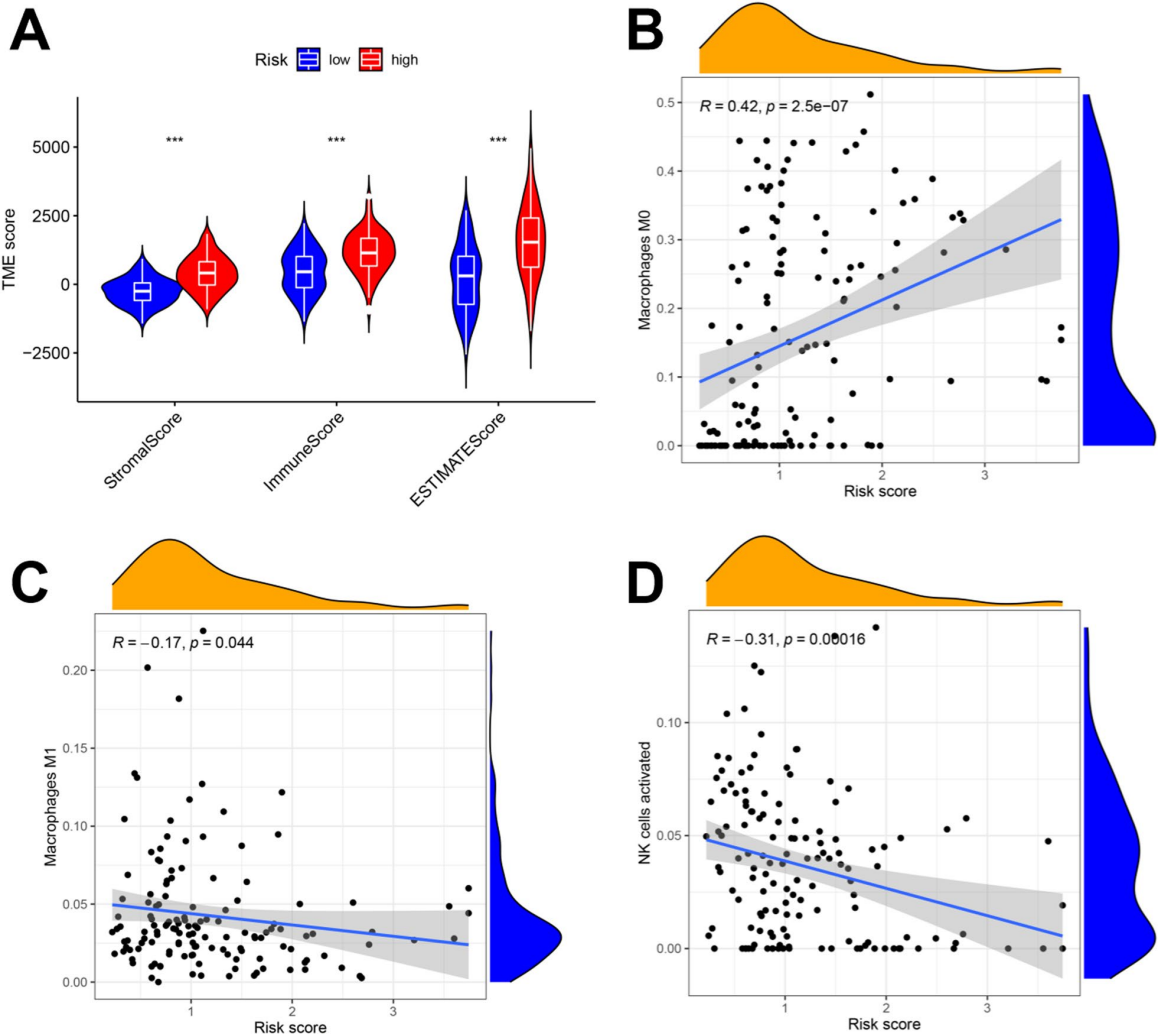
Prognostic risk score was related to TME scores and immune cell infiltration. **A** The prognostic risk score was positively correlated with the TME score. **B**–**D** The prognostic risk score was positively correlated with M0 macrophages infiltration; the prognostic risk score was negatively correlated with M1 macrophages and activated NK cells infiltration

In Fig. 2, the GSVA, GO analysis and KEGG analysis results indicated that immune-related physiological functions and pathways were markedly different in CRG cluster A and B. Therefore, we further investigated the correlations of the GBM prognostic risk model with TME scores and immune cell infiltration, and the observations showed that the TME scores were increased in the high-risk group compared with the low-risk group (Fig. 4A). The patient risk score was positively correlated with M0 macrophage infiltration and negatively correlated with M1 macrophage and activated NK cell infiltration (Fig. 4B–D). The M0 phenotype is considered an attenuated M2 phenotype, which is associated with tumor immunosuppression [24]. M1 macrophages and activated NK cells can inhibit tumor progression [25, 26]. In summary, these observations indicated that GBM patients with high risk scores were more likely to have an immunosuppressive microenvironment.

### The prognostic risk model was related to the IDH status of GBM patients

**Fig. 5:**
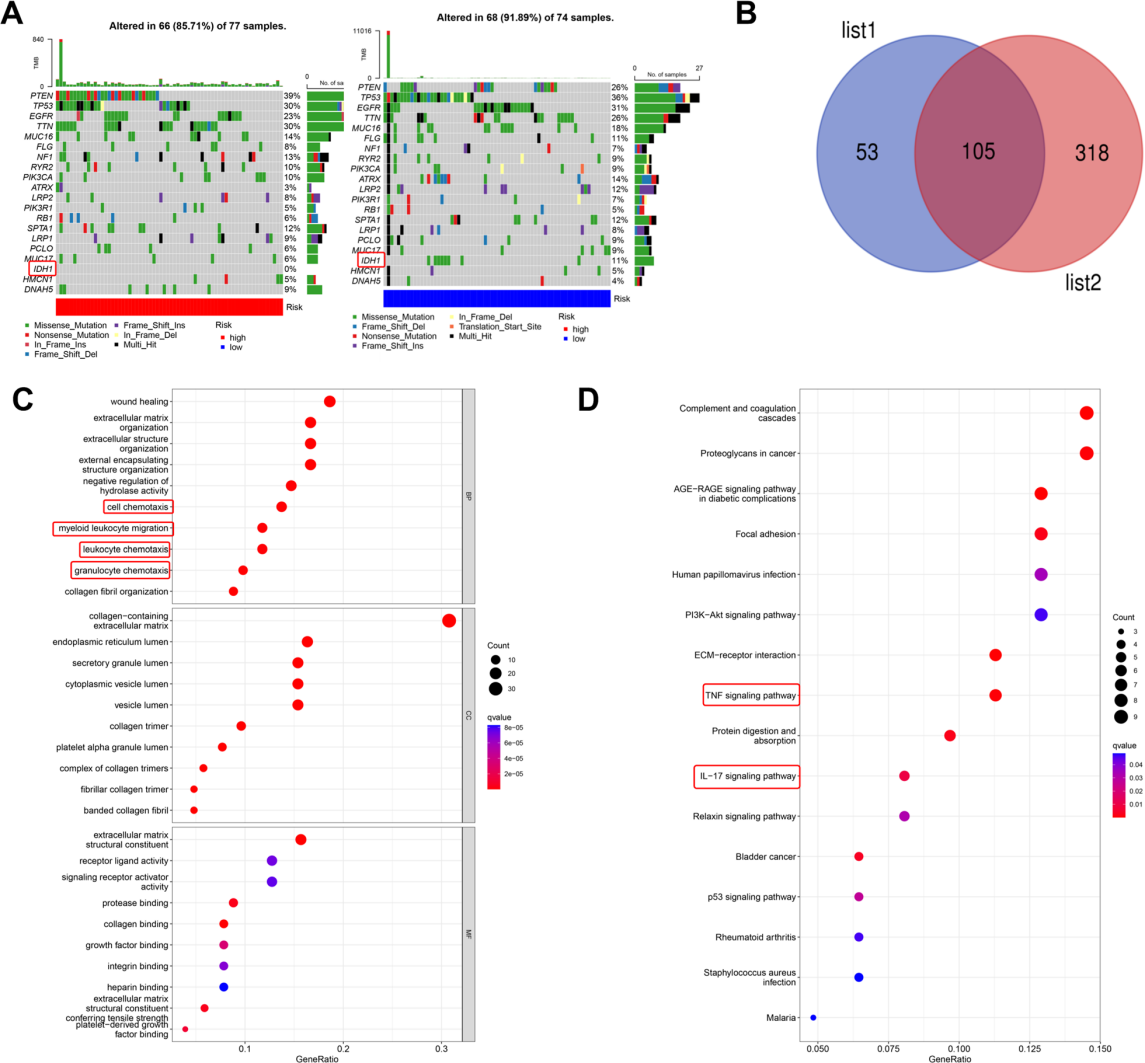
Correlation analysis of the prognostic risk model with IDH status of GBM patients. **A** The waterfall plot revealed the difference in tumor mutational burden between the high- and low-risk groups. **B** The highly expressed genes in IDH wild-type GBM and 158 prognostic high-risk genes were cross-analyzed with a Venn diagram; finally, we obtained 105 high-risk genes. List 1: 158 prognostic high-risk genes in GBM. List 2: Highly expressed genes in IDH wild-type GBM. **C**–**D** GO and KEGG analyses revealed the physiological functions and pathways affected by the 105 high-risk genes obtained from intersection analysis

Next, we explored the gene mutation burden in the prognostic risk model to gain further insights into the molecular biological characteristics of the high-risk and low-risk groups in the prognostic risk model. We found that the ratio of IDH mutations was zero in the high-risk group; in contrast, the ratio in the low-risk group was 11%. The results indicated that the GBM patients in the low-risk group were more likely to have IDH mutations (Fig. 5A). To further identify the high-risk genes related to IDH status in GBM, we obtained the transcriptome and clinical information of GBM patients from the Chinese Glioma Genome Atlas (CGGA) database and then analyzed the differential gene expression between IDH wild-type GBM and IDH-mutant GBM (Additional file 5: Figure S5A-B). Next, the highly expressed genes in IDH wild-type GBM were cross-analyzed with 158 high-risk genes among the 210 prognostic genes previously obtained in result 3, and 105 high-risk genes were obtained (Fig. 5B). Then, we determined the correlation of these 105 risk genes with GBM IDH status, and the expression of these 105 genes was higher in IDH wild-type GBM than in IDH-mutant GBM (Additional file 5: Figure S5C). Subsequently, GO and KEGG enrichment analyses were used to determine the functions of the 105 high-risk genes, and the results showed that immune-related biological processes (BPs) (cell chemotaxis, myeloid leukocyte migration, leukocyte chemotaxis and granulocyte chemotaxis) and pathways (TNF signaling pathway and IL-17 signaling pathway) were markedly regulated by these 105 genes (Fig. 5C–D). These results revealed that the 105 genes were closely involved in IDH status and immune regulation in GBM.

**Fig. 6:**
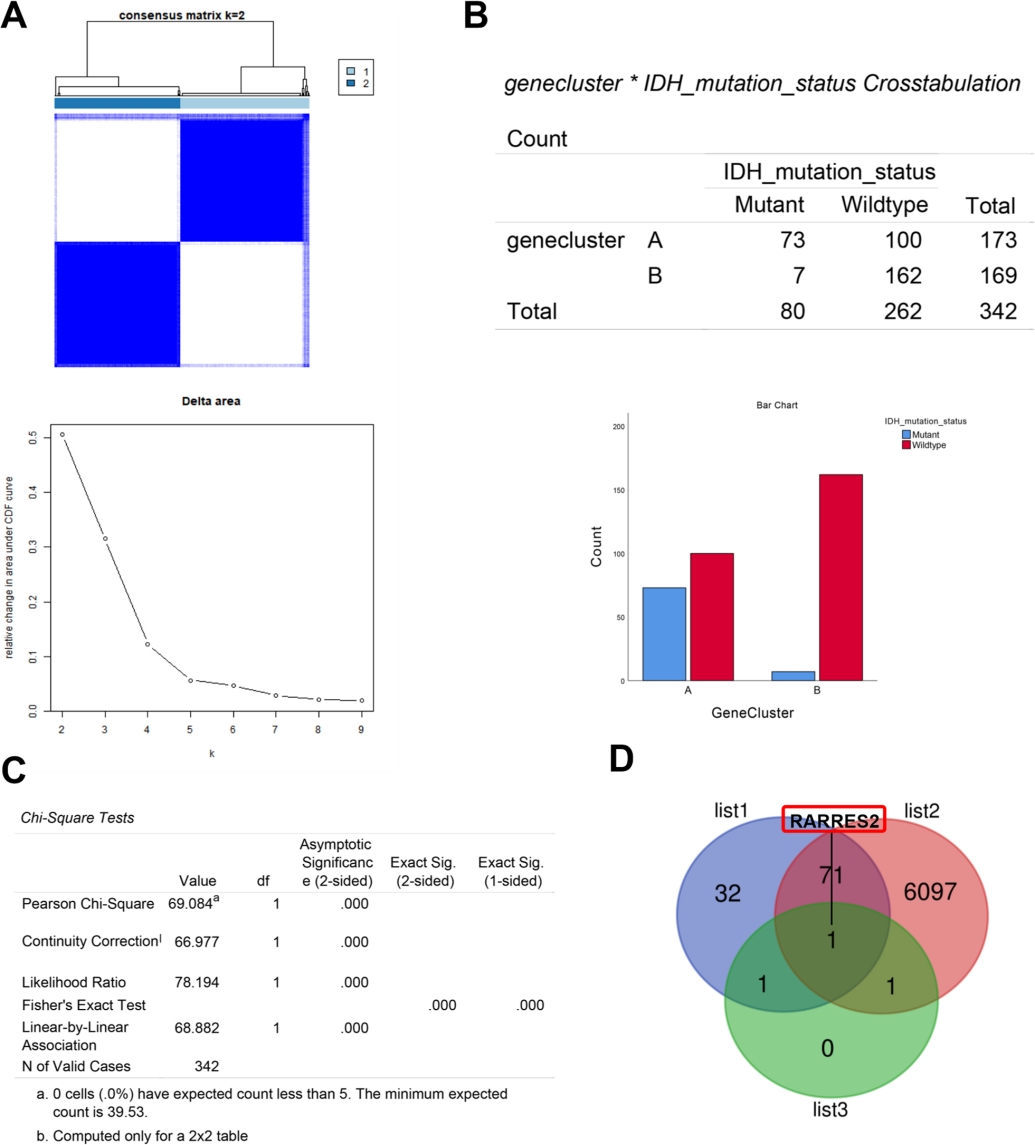
Cluster analysis was used to obtain the target genes associated with GBM IDH status. **A** Cluster analysis divided the GBM patients into two subgroups (k = 2) according to the transcriptome of 105 high-risk genes. **B**–**C** Chi-square tests were performed for subgroups A and B. **D** The genes in list 1, list 2 and list 3 were utilized for intersection analysis with a Venn diagram. List 1: 105 genes highly expressed in subgroup B; list 2: GBM-related genes obtained from the GeneCards and OMIM databases; list 3: high-risk genes used to construct the prognostic risk model

To further demonstrate the association of these 105 genes with the IDH status and prognosis of GBM, GBM patients from the CGGA database were cluster analyzed according to the expression of 105 genes, and GBM patients were further divided into two subgroups (Fig. 6A). Next, the correlations of the subgroups with the survival time and IDH status of GBM were analyzed. We found that the survival time of subgroup B was shorter than that of subgroup A, and GBM in subgroup B was more inclined to be IDH wild-type GBM (Additional file 5: Figure S5D, Fig. 6B–C), which is also consistent with the worse prognosis of IDH wild-type glioma than IDH-mutant glioma [27]. Meanwhile, the expression of 105 genes in subgroups A and B was further analyzed, and the results showed the expression of 105 genes in subgroup B was increased relative to subgroup A (Additional file 5: Figure S5E). This result again suggested that these 105 genes were inextricably related to the prognosis and IDH status of GBM patients. Next, to identify crucial genes that influence the IDH status and prognosis of GBM, the 105 genes highly expressed in subgroup B, GBM-related genes obtained from GeneCards and OMIM database and three high-risk genes for constructing a prognostic risk model (MMP19, G0S2 and RARRES2) were used for intersection analysis; ultimately, RARRES2 was screened (Fig. 6D). Taken together, these results indicated that RARRES2 can serve as an indicator of GBM prognosis and IDH status.

### RARRES2 may act as a therapeutic target for GBM, especially IDH wild-type GBM

**Fig. 7:**
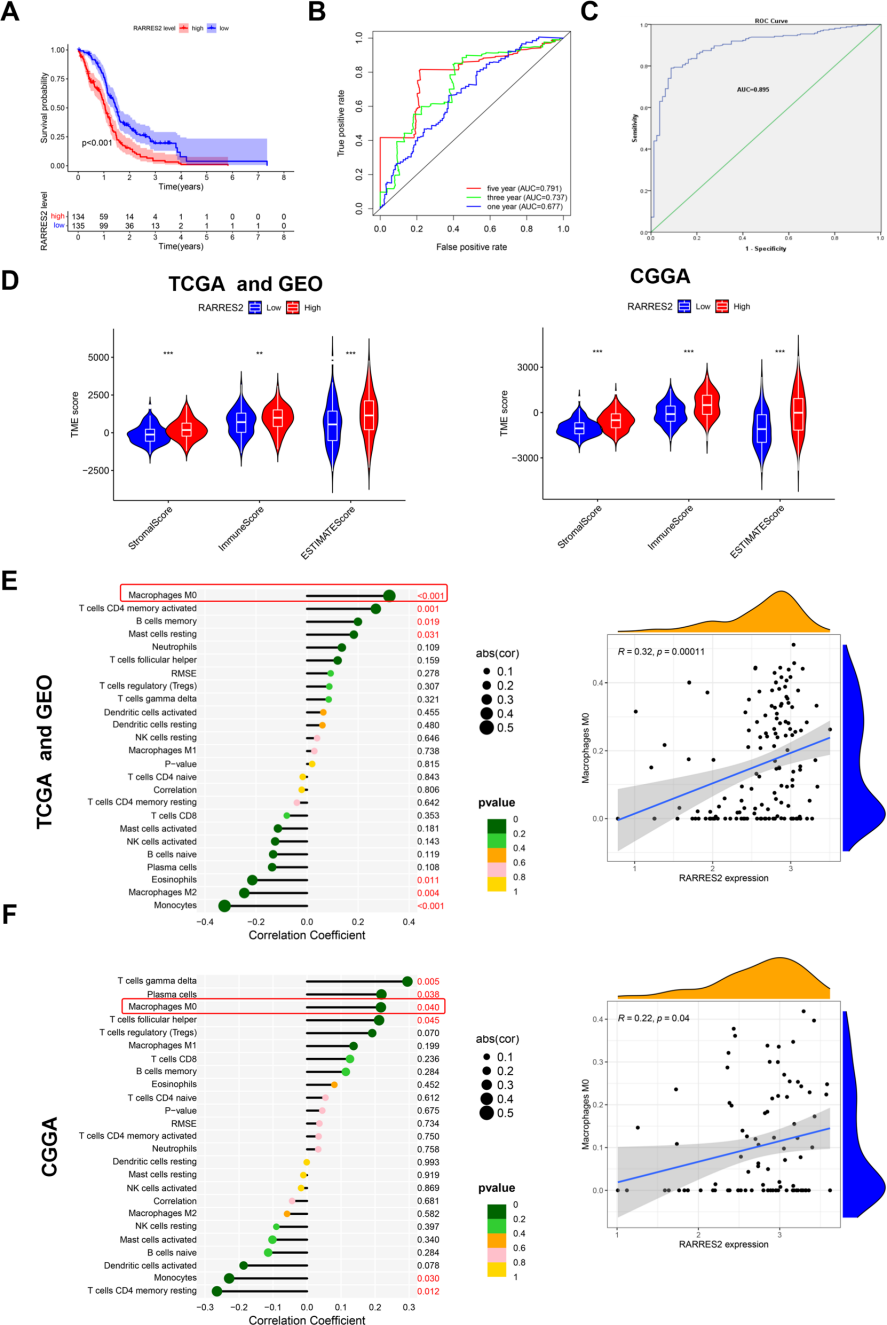
RARRES2 was associated with GBM prognosis, IDH status and immune cell infiltration. **A** Kaplan‒Meier survival analysis revealed the correlation of RARRES2 expression with GBM patient survival. **B** ROC analysis to predict the survival rates at 1, 3, and 5 years according to RARRES2 expression. **C** The ROC curve revealed the accuracy of predicting GBM IDH status by RARRES2 expression. **D** RARRES2 expression in GBM was positively correlated with the TME score. **E**–**F** RARRES2 expression in GBM was positively correlated with M0 macrophage infiltration

To further confirm the possibility of RARRES2 as a target for GBM treatment, we analyzed the relationship between RARRES2 expression and GBM survival time. The Kaplan‒Meier survival analysis results indicated that the expression of RARRES2 was negatively correlated with the survival time of GBM (Fig. 7A). The subsequent ROC analysis showed that the AUC value was greater than or equal to 0.7, suggesting that RARRES2 can serve as a predictor of GBM patient prognosis (1, 3, and 5 years) (Fig. 7B). Next, we evaluated the accuracy of RARRES2 expression in predicting IDH status in GBM. The ROC analysis results showed that the AUC was 0.895, indicating that patients with high expression of RARRES2 tend to have IDH wild-type GBM (Fig. 7C). Specific immune cell infiltration is the key barrier of immunotherapy in a variety of tumors [28-31]; therefore, the correlation of RARRES2 expression with the immune microenvironment was further explored. The results revealed that the expression of RARRES2 is positively correlated with the TME score in GBM, and high RARRES2 expression can recruit M0 macrophages to infiltrate the GBM microenvironment. We also found that RARRES2 expression was positively correlated with the expression of most checkpoints, such as PD-L1 (CD274) (Fig. 7D–F Additional file 5: Figure S5F). These observations suggested that RARRES2 expression was closely related to prognosis, IDH status and the formation of an immunosuppressive microenvironment in GBM.

**Fig. 8:**
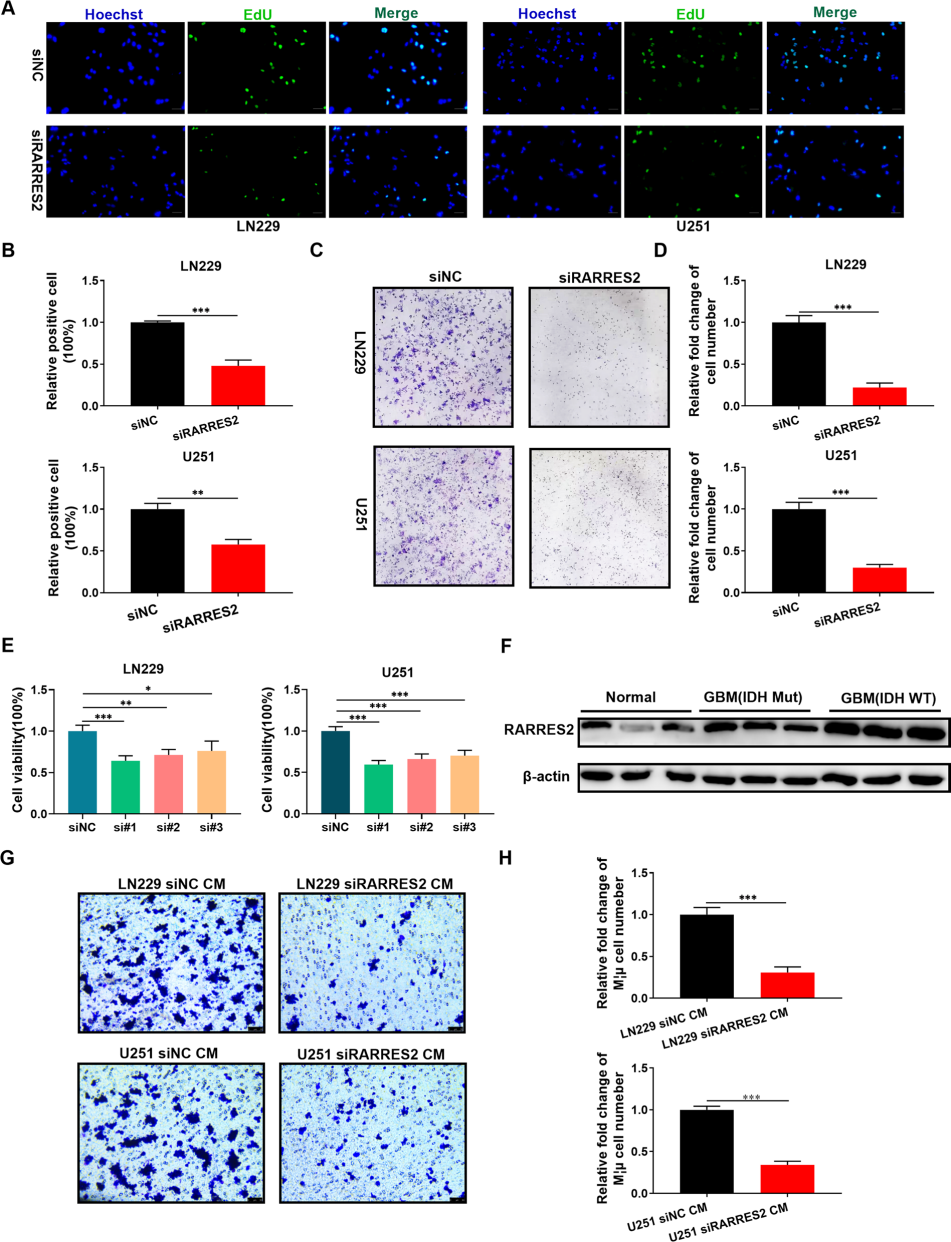
Targeting RARRES2 inhibits GBM progression and immune cell infiltration. **A**–**B** U251 and LN229 glioma cells were treated with siRARRES2 for 48 h. The EdU assay was used to detect glioma cell proliferation ability (bar: 100 µm). **C**–**D** U251 and LN229 glioma cells were treated with siRARRES2 for 48 h, and a colony formation assay was used to detect the colony formation ability of glioma cells. **E** U251 and LN229 glioma cells were treated with siRARRES2 for 48 h, and MTT assays were used to detect glioma cell viability. **F** The relative protein expression of RARRES2 in normal brain tissue and IDH (Mut) and IDH (WT) GBM tissues was assessed by Western blotting. Normal: normal brain tissue. IDH(Mut): IDH-mutant GBM tissue. IDH(WT): IDH wild-type GBM tissue. **G**–**H** The conditioned media was used to culture macrophages for 48 h, and the invasion ability of macrophages was analyzed by transwell assay (bar: 50 µm). Error bars: mean ± SD. *P < 0.05, **P < 0.01, ***P < 0.001

Next, we explored the effects of targeting RARRES2 on GBM. siRNA was used to knockdown RARRES2 in U251 and LN229 glioma cell lines, and the efficiency of gene knockout was shown in Additional file 5: Figure S5G. The proliferation ability of GBM (U251 and LN229) cells treated with RARRES2 siRNA was detected by EdU assay, and we found that RARRES2 siRNA treatment decreased the green fluorescence intensity, suggesting that the proliferation ability of GBM cells was inhibited by siRARRES2 (Fig. 8A–B). Moreover, a colony formation assay was performed to detect the effects of siRARRES2 on GBM, and the results showed that siRARRES2 also had an inhibitory effect on GBM colony formation ability (Fig. 8C–D). Subsequently, the MTT results showed that RARRES2 knockdown significantly reduced GBM (U251 and LN229) cell viability (Fig. 8E). These results indicated that targeting RARRES2 exerts an antitumor effect on GBM. To further assess the correlation of RARRES2 expression with IDH status in GBM, Western blotting results showed that the relative protein level of RARRES2 in IDH wild-type GBM patients was higher than that in IDH-mutant GBM patients (Fig. 8F). These results and those shown in 7C fully demonstrated that the expression level of RARRES2 was correlated with GBM IDH status. To demonstrate that RARRES2 expression was associated with macrophage infiltration in GBM, Transwell experiments further verified that the coculture of glioma cell lines (U251 and LN229) in which RARRES2 was knocked down with macrophages could significantly reduce macrophage infiltration (Fig. 8G–H).

Taken together, the above results indicate that RARRES2 is associated with GBM prognosis and IDH status, and RARRES2 can serve as an immunotherapy target for GBM treatment, especially IDH wild-type GBM.

## Discussion

Mounting evidences have confirmed that the copper signaling pathway is associated with the biological behaviors of malignancy, including angiogenesis, metastasis, and proliferation. However, more mechanistic studies are still needed to link copper metabolism to copper-dependent disease vulnerability, particularly in cancer, which will help translate basic research on copper chemistry and biology into potential clinical therapies [7]. The proposed mode of cuproptosis systematically elucidates the relationships between copper metabolism and cell death and mitochondrial disorders [5]; moreover, the conception provides a theoretical foundation for exploring the role of CRGs in tumors. Given that the effect of cuproptosis in GBM is poorly understood, in the present study, 64 CRGs were screened for systematic research. After subsequent in-depth analysis, the GBM prognostic risk model was finally constructed with three genes (MMP19, G0S2 and RARRES2). Finally, we confirmed that RARRES2 related to CRG clusters could be used as a crucial target gene for GBM prognosis evaluation, IDH status prediction and immunotherapy.

Under normal conditions, copper homeostasis can maintain the normal operation of various biological processes [32]. Dysregulation of copper homeostasis can induce cell death, which is called cuproptosis [5]. Copper metabolism is dynamic in a variety of tumors. For example, abnormal copper accumulation may promote the transformation of malignant biological behaviors in hepatocellular carcinoma [33]; moreover, to support unrestricted proliferation, cancer cells, such as lung, oral, and thyroid cancers, have a stronger need for copper than healthy cells [7]. However, it is not clear whether copper metabolism is also abnormally regulated in GBM cells, and studies have confirmed that changes in copper transcription levels are important for tumor progression [34]. Therefore, the expression of CRGs was analyzed in our study, and the results confirmed that the expression of CRGs significantly differed between GBM and normal brain tissues. Further combined analysis of the TCGA and GEO databases suggested that CRGs were differentially expressed in GBM and correlated with the prognosis of GBM patients. In addition, research has reported abnormal regulation of copper metabolism in tumor tissues relative to healthy tissues [22]. Together, these evidences confirmed that copper metabolism was abnormally regulated in GBM.

Given the abnormal expression of CRGs in GBM, GBM was clustered into CRG cluster A and B, and then 210 genes related to the prognosis of GBM were screened from the CRG clusters. Next, 210 genes were further analyzed by cluster analysis, LASSO regression and Cox analysis. Ultimately, the prognostic risk model was established, which was composed of MMP19, G0S2 and RARRES2. MMP19 (matrix metallopeptidase 19), a relatively new member of the MMP family, is highly expressed in non-small cell lung cancer (NSCLC) and is associated with NSCLC progression [35]. In gallbladder carcinoma (GBC), MMP19 can stabilize the epithelial–mesenchymal transition (EMT) by increasing Axl expression [36]. G0S2 (G0/G1 switch 2) is upregulated and related to radiotherapy resistance in GBM [37]. RARRES2 (retinoic acid receptor responder 2) has different expression patterns in different tumors; for example, in acute myeloid leukemia (AML) and breast cancer, RARRES2 expression is downregulated [38]; however, RARRES2 is overexpressed in oral squamous cell carcinoma, and the overexpression of RARRES2 is associated with angiogenesis and poor prognosis of tumors [39]. In brief, all three key genes are closely related to tumor progression. Further analysis of the prognostic risk model showed that the GBM patients in the high-risk group had higher expression of three (MMP19, G0S2 and RARRES2), higher risk scores and shorter survival times. The correlation analysis of CRG clusters, gene clusters, and prognostic factor risk scores and ROC analysis indicated that the constructed scoring system has an accurate predictive ability for GBM prognosis.

In neuroblastoma, elevated intracellular copper concentrations can regulate the expression of PD-L1, thereby causing tumor immune evasion [11]. Moreover, copper has a strong regulatory effect on immune processes [40]. In addition, the infiltration of immune cells is considered an indicator of poor prognosis in glioma [24]. Therefore, further research on the correlation of CRG clusters with the immune microenvironment could enhance the understanding of anti-GBM immunotherapy and provide guidance for the development of new immunotherapeutic targets in GBM. In the present research, we conducted GO and KEGG enrichment analyses on the DEGs in the CRG clusters, and found that the differentially expressed genes obtained from CRG clusters can significantly affect immune-related functions and pathways in GBM. Further correlation analysis between the prognostic risk model and immune microenvironment showed that the TME score of GBM patients in the high-risk subgroup was significantly higher than that of GBM patients in the low-risk subgroup. Moreover, patient risk scores were negatively correlated with M1 macrophage and activated NK cell infiltration and positively correlated with M0 macrophage infiltration. M1 macrophages can produce proinflammatory factors, which are thought to be associated with tumor suppression [25]. The M0 phenotype is considered the attenuated M2 phenotype, while the M2 phenotype is considered associated with tumor angiogenesis and the formation of a tumor immunosuppressive microenvironment [24]. Activated NK cells can restrain tumor growth and spread [26]; therefore, these immune cells are involved in the formation of the tumor immune microenvironment and tumor progression. Considering the above correlations between the CRG clusters and prognostic characteristic genes and the immune microenvironment, the findings further suggested that the CRG clusters are closely related to the formation of an immunosuppressive microenvironment and prognosis in GBM.

TMB can help predict the therapeutic response to immunotherapy [41]. The prognostic risk model constructed in our study was also associated with immune cell infiltration, the formation of TME and GBM prognosis; therefore, TMB was further evaluated in the prognostic risk model, and we found that patients in the high-risk subgroup tended to have IDH wild-type GBM, which is consistent with the worse prognosis of patients with IDH wild-type gliomas [27]. To further screen the crucial genes affecting the prognosis and IDH status of GBM in the prognostic risk model, in-depth analysis was performed, and the results of survival analysis and ROC analysis also confirmed that abnormal RARRES2 overexpression was associated with the poor prognosis of GBM patients. The results of ROC analysis suggested that the expression of RARRES2 had high accuracy in predicting IDH status (AUC = 0.895) in GBM. Moreover, we further evaluated the therapeutic value of targeting RARRES2 in GBM and the ability of RARRES2 to predict GBM IDH status. The results indicated that RARRES2 knockdown can significantly reduce GBM cell viability and proliferation activity, and the protein expression level of RARRES2 was significantly correlated with IDH status in GBM patients. Although RARRES2 has different expression patterns in different tumors, RARRES2 can promote GBM mesenchymal properties by inhibiting the ubiquitin‒proteasome degradation of CMKLR1 [42], which indicates that RARRES2 can support tumor progression in GBM. Our results also demonstrated that the expression of RARRES2 in GBM was positively correlated with TME scores and M0 macrophage infiltration and positively correlated with the expression of immune checkpoints, such as PD-L1 (CD274). Moreover, the subsequent transwell assay showed that targeting GBM RARRES2 could decrease macrophage infiltration, which also suggested that RARRES2 was associated with the formation of an immunosuppressive microenvironment in GBM. Overall, RARRES2 is likely to be a target of IDH wild-type GBM immunotherapy.

The innovativeness of this study is mainly that we innovatively found that abnormal copper metabolism is associated with the IDH status in GBM through various analysis methods, including tumor mutational burden analysis, intersection analysis, and prognostic risk model construction. The high-risk group of GBM patients in the prognostic risk model tended to have IDH wild-type GBM and was more prone to have immunosuppressive phenotypes. Meanwhile, given that IDH wild-type GBM patients have a worse prognosis and lack of effective therapeutic strategies, the correlations among copper metabolism, IDH status and the immune microenvironment in GBM were further analyzed comprehensively, and the observations revealed that RARRES2 expression is different in GBM CRG clusters. Further studies showed that abnormal RARRES2 expression is correlated with IDH status and immune cell infiltration in GBM patients. Finally, we speculate that RARRES2 obtained from GBM CRG clusters may be a target of immunotherapy for IDH wild-type GBM patients. This study is a further expansion of previous articles on copper metabolism in GBM as well as on RARRES2, which provides a new strategy for the individualized therapy of IDH wild-type GBM patients. However, our study still has some limitations. First, the experimental data were derived from public databases, which inevitably leads to data deviation. Second, more clinical pathological specimens and experiments are needed to verify the expression and physiological functions of target genes in vivo and in vitro, which is our direction for future research.

In summary, we established a prognostic risk model for GBM patients through a comprehensive and in-depth analysis of CRG clusters and confirmed that the prognostic risk model is related to the prognosis, TMB, and immune microenvironment of GBM patients. Moreover, further analysis and experiments confirmed that RARRES2 could serve as a novel target for GBM immunotherapy, particularly in IDH wild-type GBM.

## Supplementary Information

**Supplementary Figure 1.** The Kaplan‒Meier survival analysis of 20 CRGs associated with GBM survival and HR risk.

**Supplementary Figure 2.** The PCA, Kaplan‒Meier survival analysis and differentially expressed genes analysis of CRG cluster A and B.

**Supplementary Figure 3.**  The Kaplan‒Meier survival analysis of gene cluster A and B.

**Supplementary Figure 4.**  The association of prognostic risk model with CRG cluster and gene cluster.

**Supplementary Figure 5.** The joint analysis of GBM IDH status-related genes and 210 GBM prognosis-related genes.

## Data Availability

The publicly available datasets included in this research could be found in Material and Methods.
