# Supplementary figures and images for: Targeting copper death genotyping associated gene RARRES2 suppresses glioblastoma progression and macrophages infiltration
Source: Cancer Cell Int. 2023 May 29;23:105. doi: 10.1186/s12935-023-02950-6 (PMC10226210; doi:10.1186/s12935-023-02950-6)

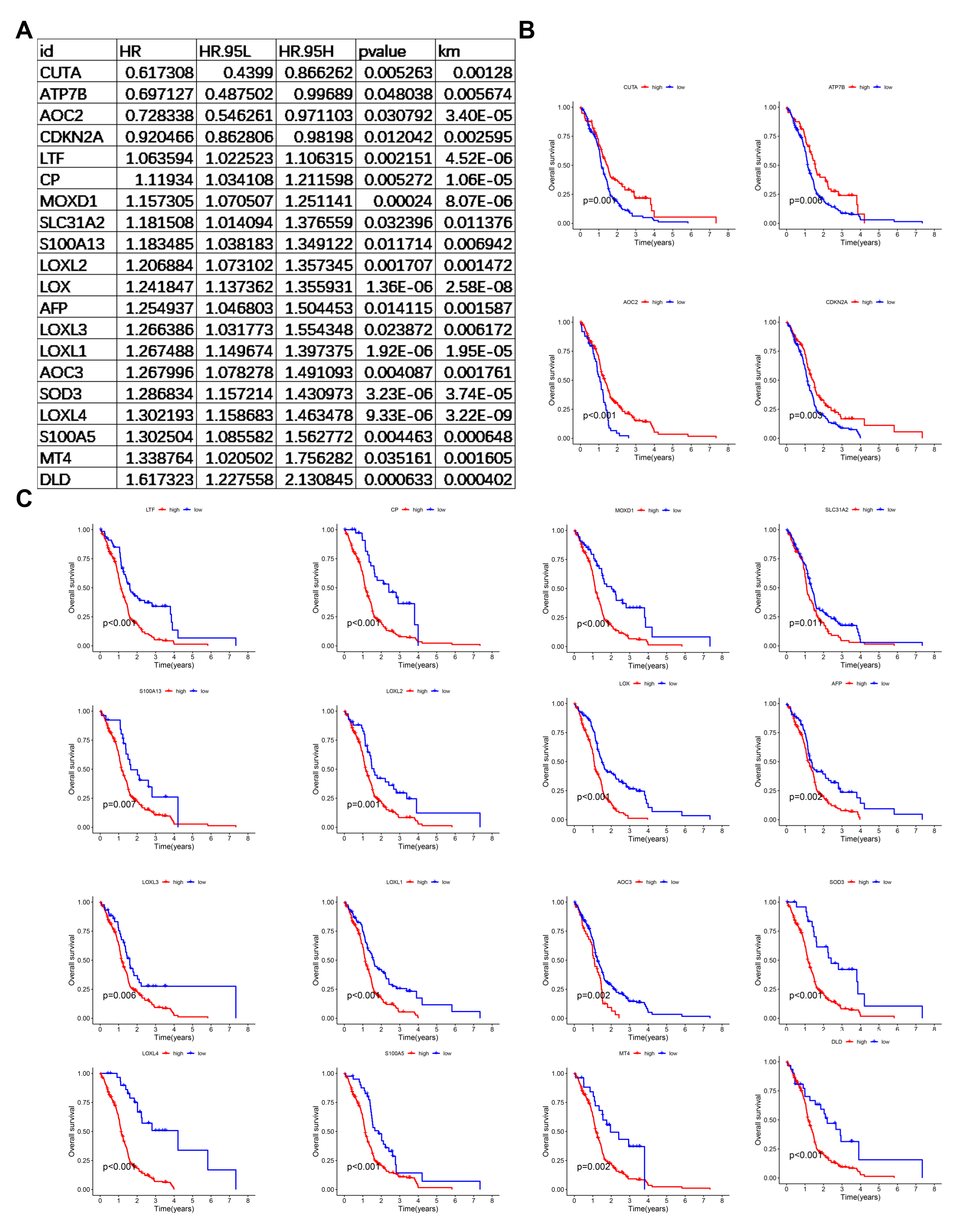

Supplement: Supplementary file 1 — Supplementary Figure 1. The Kaplan‒Meier survival analysis of 20 CRGs associated with GBM survival and HR risk. [file 12935_2023_2950_MOESM1_ESM.tif]

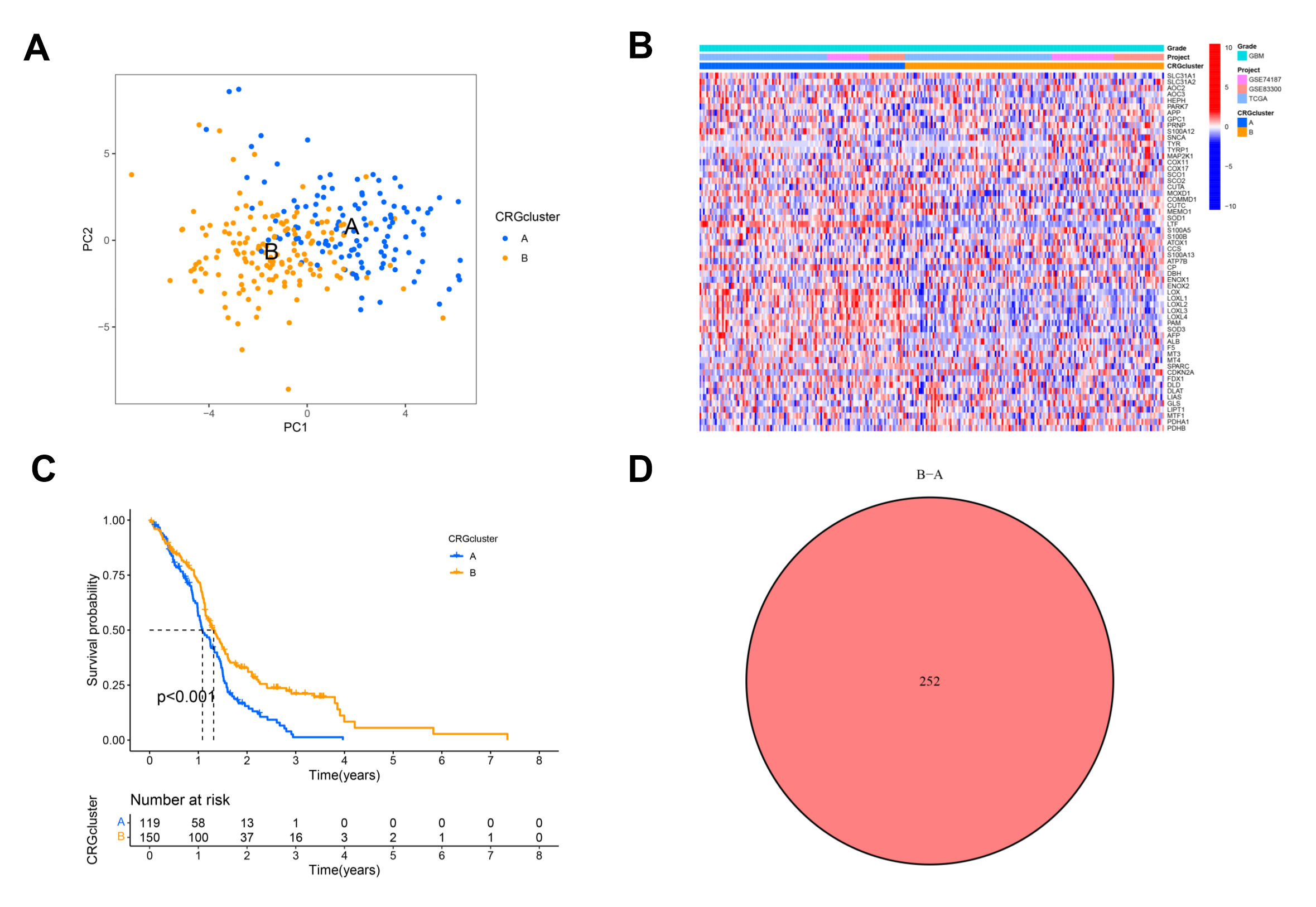

Supplement: Supplementary file 2 — Supplementary Figure 2. The PCA, Kaplan‒Meier survival analysis and differentially expressed genes analysis of CRG cluster A and B. [file 12935_2023_2950_MOESM2_ESM.tif]

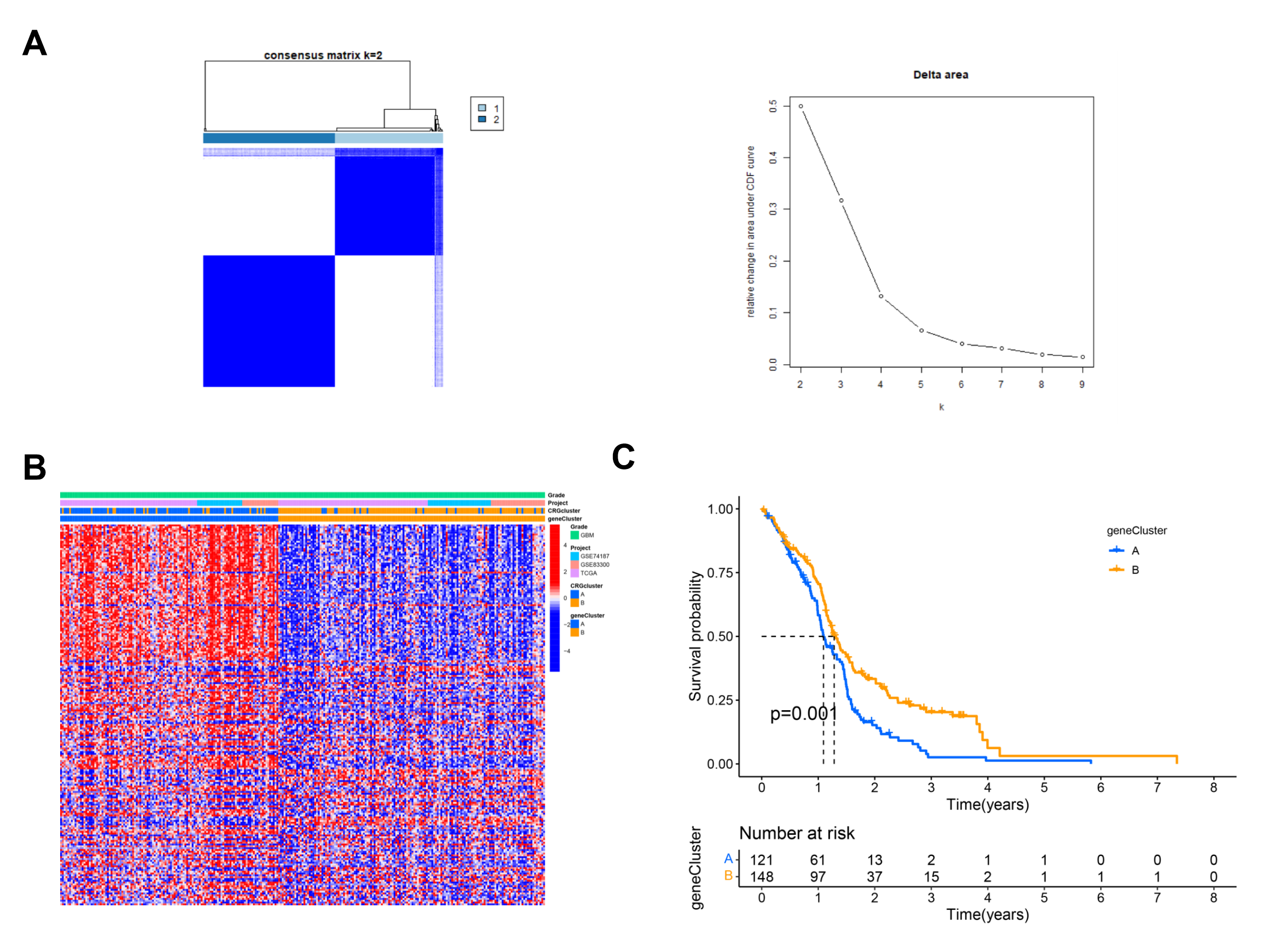

Supplement: Supplementary file 3 — Supplementary Figure 3. The Kaplan‒Meier survival analysis of gene cluster A and B. [file 12935_2023_2950_MOESM3_ESM.tif]

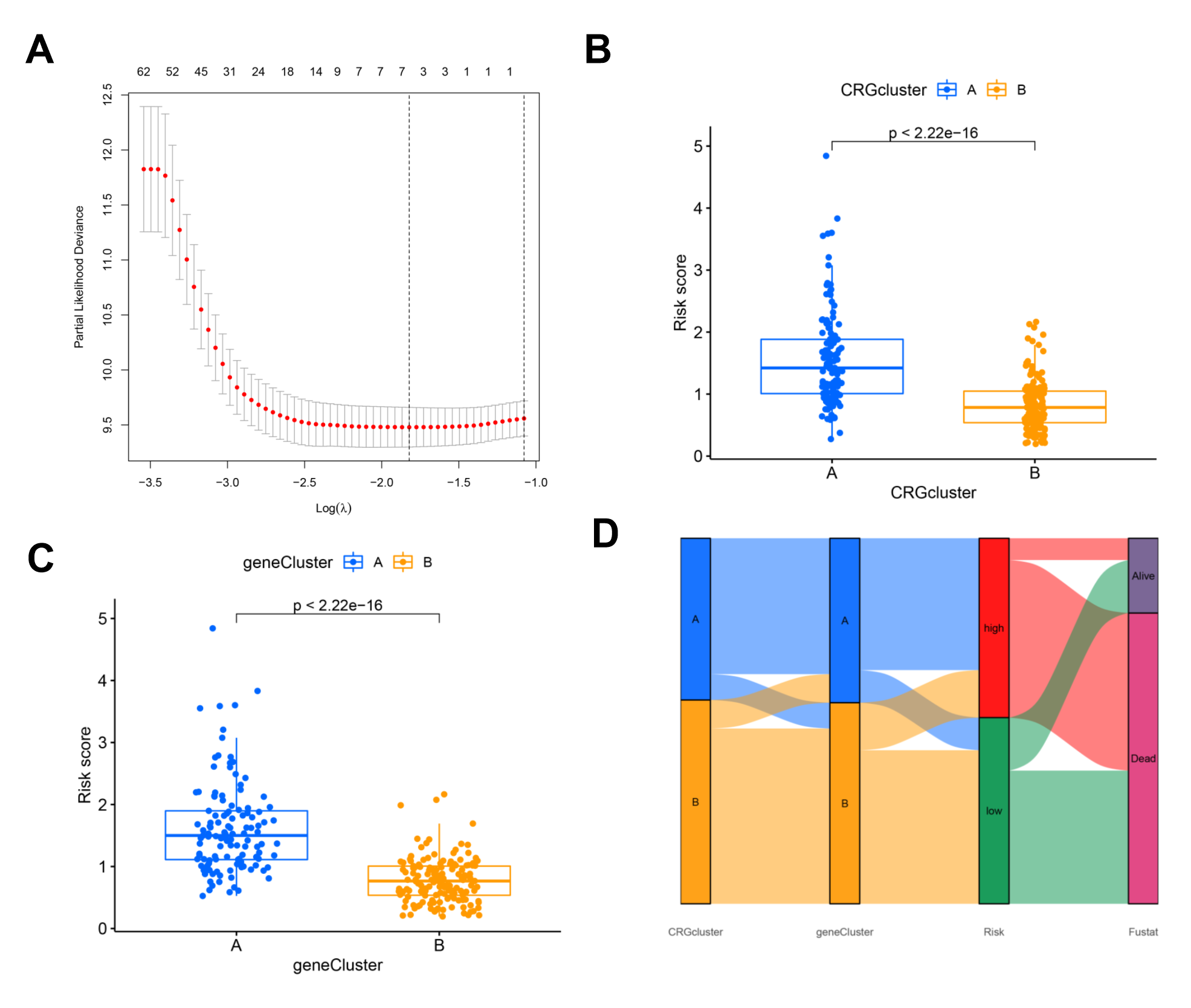

Supplement: Supplementary file 4 — Supplementary Figure 4. The association of prognostic risk model with CRG cluster and gene cluster. [file 12935_2023_2950_MOESM4_ESM.tif]

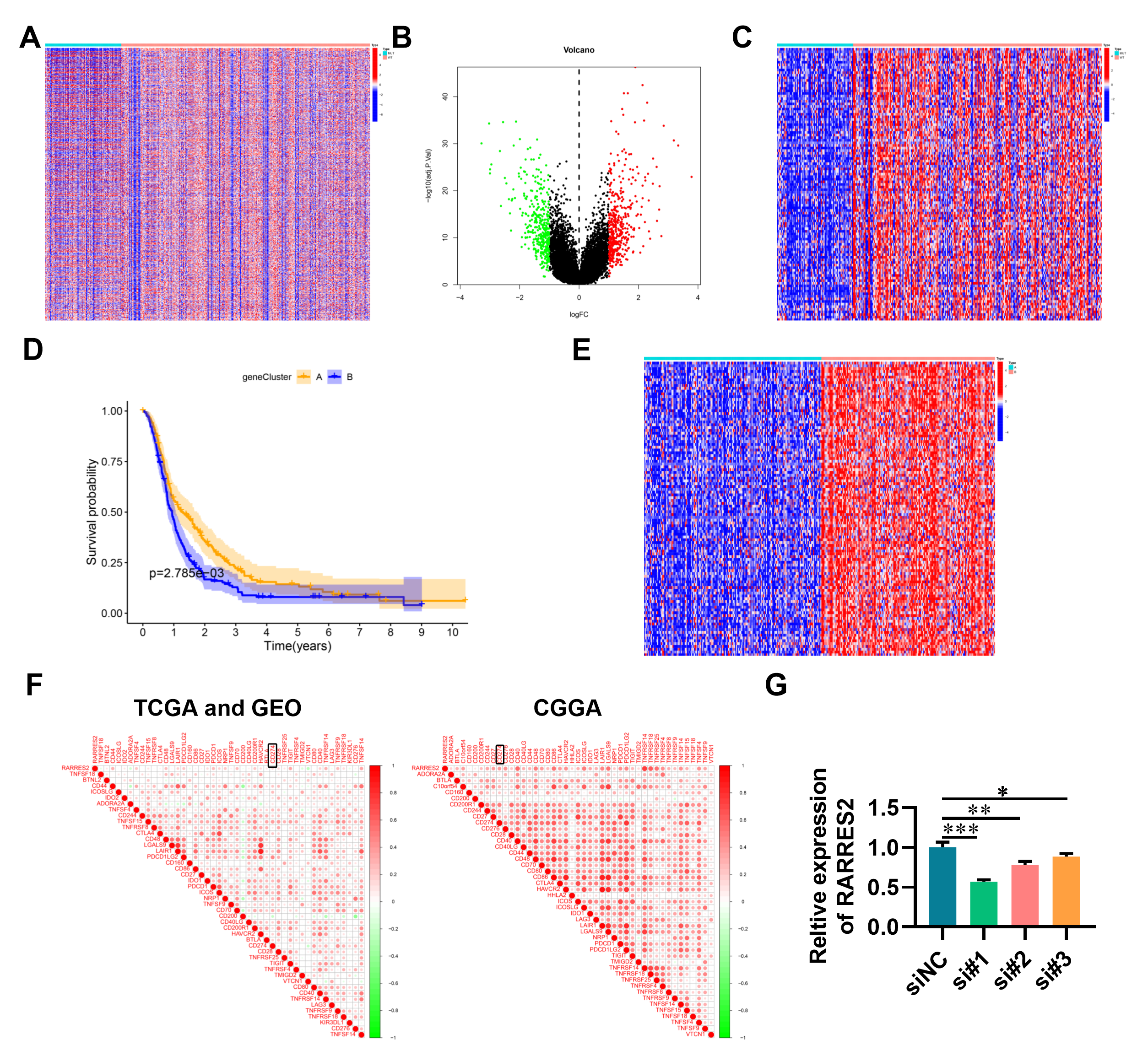

Supplement: Supplementary file 5 — Supplementary Figure 5. The joint analysis of GBM IDH status-related genes and 210 GBM prognosis-related genes. [file 12935_2023_2950_MOESM5_ESM.tif]
